# Schisandrol A Exhibits Estrogenic Activity via Estrogen Receptor α-Dependent Signaling Pathway in Estrogen Receptor-Positive Breast Cancer Cells

**DOI:** 10.3390/pharmaceutics13071082

**Published:** 2021-07-15

**Authors:** Dahae Lee, Young-Mi Kim, Young-Won Chin, Ki Sung Kang

**Affiliations:** 1 College of Korean Medicine, Gachon University, Seongnam 13120, Korea; pjsldh@gachon.ac.kr; 2 College of Pharmacy and Research Institute of Pharmaceutical Sciences, Seoul National University, 1, Gwanak-ro, Gwanak-gu, Seoul 08826, Korea; 0210121@hanmail.net

**Keywords:** phytoestrogens, estrogen receptor, schisandrol A

## Abstract

The aim of this study was to examine the estrogen-like effects of gentiopicroside, macelignan, γ-mangostin, and three lignans (schisandrol A, schisandrol B, and schisandrin C), and their possible mechanism of action. Their effects on the proliferation of the estrogen receptor (ER)-positive breast cancer cell line (MCF-7) were evaluated using Ez-Cytox reagents. The expression of extracellular signal-regulated kinase (ERK), phosphatidylinositol 3-kinase (PI3K), AKT, and estrogen receptor α (ERα) was measured by performing Western blot analysis. 17β-estradiol (E2), also known as estradiol, is an estrogen steroid and was used as a positive control. ICI 182,780 (ICI), an ER antagonist, was used to block the ER function. Our results showed that, except for gentiopicroside, all the compounds promoted proliferation of MCF-7 cells, with schisandrol A being the most effective; this effect was better than that of E2 and was mitigated by ICI. Consistently, the expression of ERK, PI3K, AKT, and ERα increased following treatment with schisandrol A; this effect was slightly better than that of E2 and was mitigated by ICI. Taken together, the ERα induction via the PI3K/AKT and ERK signaling pathways may be a potential mechanism underlying the estrogen-like effects of schisandrol A. This study provides an experimental basis for the application of schisandrol A as a phytoestrogen for the prevention of menopausal symptoms.

## 1. Introduction

The normal physiological functions of estrogen, a steroid hormone, are important for the development of the female reductive system [1]. When estrogen binds to the estrogen receptor (ER), its biological effects are exerted. In the nucleus, these receptors bind to DNA as members of ligand-activated transcription factors [2]. Estrogen promotes the proliferation of both normal and tumorous breast cells [3]. It plays an important role in the development of postmenopausal diseases, including hormone-dependent cancer, osteoporosis, and cardiovascular diseases. Many of these diseases are caused by a deficiency in endogenous estrogen [4]. Traditionally, estrogen replacement therapy is used to alleviate postmenopausal symptoms. However, long-term use of these therapies often causes side effects, such as hypertension, dementia, and breast cancer [5,6].

Thus, effective and less toxic alternatives, such as plant-derived estrogen, phtyoestrogens, are attracting attention for the prevention and treatment of postmenopausal symptoms. As a result, research on effective and low-toxicity alternatives, such as estro phytoestrogen from a variety of natural sources, has gained momentum [7,8,9]. Phytoestrogens derived from a variety of natural sources have potential for applications in the prevention and treatment of postmenopausal symptoms. The estrogen-like effects of plant extracts and isolated compounds, including lignans, coumestans, isoflavonoids, stilbenes, and flavonoids, have also been reported [10,11].

In particular, lignans, a dimeric form of two phenylpropanoids that is widely found in the plant kingdom, including in fruits (apricot, grapefruit, and pear), grains (barely, oat, and wheat), seeds (flaxseed, sesame seed, and sunflower seed), vegetables (cucumber, green bean, and potato), and medicinal plants, have been demonstrated to possess a variety of biological activities (anti-inflammatory, antioxidant, antiviral, antitumor, and immuno-suppressive effects), as well as protection against cardiovascular diseases [12,13]. Moreover, the effect of lignans on the risk of postmenopausal breast cancer was investigated in clinical studies [10,14,15,16]. In postmenopausal women, intake of lignans, namely pinoresinol, secoisolariciresinol, matairesinol, and lariciresinol, significantly reduces the risk of postmenopausal, ER-positive breast cancer [15]. A previous cell-based study showed that lignans, namely enterodiol, 7-hydroxymatairesinol, enterolactone, and arctigenin, show estrogen activity in yeast cells expressing human estrogen receptor α (ERα) [17]. However, compared to isoflavonoids that have been intensively investigated in phytoestrogen studies, extensive research on lignans is lacking. In addition, very few cell-based experiments using ER-positive breast cancer cell lines with high levels of estrogen signaling have been used to investigate the estrogen-like effect of lignans [11].

The literature studying the estrogen-like effect of lignans makes use of various in vitro bioassays, including the yeast-based reporter gene assay and breast cancer cell-based assay systems [18]. However, the sensitivity of the yeast-based reporter gene assay is commonly lower than that of breast cancer cell-based assay systems [19]. The experimental model chosen for evaluating estrogenic activity was the E-screen assay that was developed by Soto et al. in 1995 [20]. This assay has been widely used as a quick experimental model to detect potential phytoestrogen. Herein, estrogenic activity is measured using an ER-positive breast cancer cell line (MCF-7) [21]. MCF-7 cells are frequently used because these cell lines exhibit a more consistent proliferative response to estrogen across a range of many passages [22]. In this study, we selected several compounds with different skeletons: iridoid-type (gentiopicroside), xanthone-type (γ-mangostin), and lignan-type compounds (macelignan, schisandrol A, schisandrol B, and schisandrin C), which were previously reported to regulate ERK signaling involved in cell survival, differentiation, and proliferation [23,24,25,26,27,28,29,30,31] and, for the first time, we evaluated their estrogen-like effects in MCF-7 cells. In addition, ERα expression via the PI3K/AKT and ERK signaling pathways, as potential molecular targets underlying the estrogen-like effects, was evaluated using Western blotting techniques.

## 2. Materials and Methods

### 2.1. Plant Material

The dried bark of *Gentiana macrophylla* Pallas (*G. macrophylla*; Gentianaceae), *Myristica fragrans* Houttuyn (*M. fragrans*; Myristicaceae) seeds, and *Garcinia mangostana* L (*G. mangostana*) was purchased from an oriental market in June 2018 and identified by Dr. Hee-Sung Chae. Voucher specimens (CYWSNU-KP0020 for *G. macrophylla*, CYWSNU-KP0021 for *M. fragrans*, and CYWSNU-CP005 for *G. mangostana*) were deposited in the medicinal plant garden at Seoul National University.

### 2.2. Extraction and Isolation of Compounds

The roots of *G. macrophylla* (2.0 kg) were extracted three times with MeOH. The MeOH extract (427.65 g) was suspended in water and partitioned successively with n-hexane, CHCl_3_, EtOAc, and *n*-BuOH to give a residue of 34.70 g of CHCl_3_ fraction, 14.71 g of EtOAc fraction, 160.0 g of n-BuOH fraction, and a water-soluble fraction. The *n*-BuOH fraction (160.0 g, GMB) was chromatographed over a silica column using a gradient of increasing polarity with CHCl_3_-MeOH (100:0 to 1:1) as the solvent, and was fractioned into 12 sub-fractions (GMB1-GMB12). GMB7 (9.5 g) was subjected to RP-MPLC eluted with MeOH-water (0:100 to 80:20) to give two sub-fractions (GMB7A and GMB7B). GMB7B (7.2 g) was purified by silica gel column chromatography using CHCl_3_-MeOH (50:1 to 1:1) as the solvent and was washed with MeOH to give four sub-fractions (GMB7B1–GMB7B4), including gentiopicroside. The structure of compound was identified using ^1^H NMR and ^13^C NMR spectroscopic data.

*M. fragrans* seeds (600.0 g) were extracted with MeOH three times. The MeOH extract (114.38 g) was suspended in water and partitioned between EtOAc successively to give the residue EtOAc fraction (98.05 g, MFE). The EtOAc fraction (96.78 g, MFE) was chromatographed over a silica gel column using a gradient of n-hexane-EtOAc (100:0 to 1:1) into five sub-fractions (MFE1-MFE5). MFE3 (34.5 g) was subjected to RP-MPLC and eluted with MeOH-water (50:5 to 90:10). Macelignan was isolated as a precipitate in the MFE3C fraction. The structure of compound was identified using ^1^H NMR and ^13^C NMR spectroscopic data.

The dried pericarp of *G. mangostana* L (1.23 kg) was extracted with EtOH three times. The EtOH extract (87.1 g) was suspended in water and the solvent was partitioned with CHCl_3_, EtOAc, and *n*-BuOH, yielding 50.18 g, 2.74 g, and 18.27 g of residue, respectively. The CHCl_3_ fraction (45.93 g, GMC) was chromatographed over silica gel column using a gradient of CHCl_3_-MeOH (100:0 to 0:100) as the solvent, giving 18 sub-fractions (GMC1-GMC18). The GMC12 (4.73 g) fraction was separated using preparative RP-MPLC with 40–80% MeOH to yield γ-mangostin. The structure of compound was identified using ^1^H NMR and ^13^C NMR spectroscopic data.

**Figure 1:**
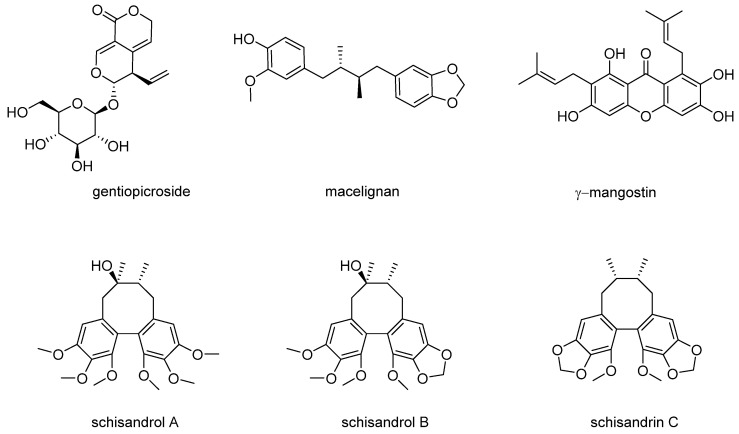
Chemical structures of compounds.

The chemicals used in the present study, schisandrol A, schisandrol B, and schisandrin C (Figure 1), were obtained from previous studies, and the ^1^H and ^13^C NMR spectroscopic data are provided in the Supplementary Materials.

### 2.3. Purity Analysis of Compounds

Purity analysis was performed on an Ultimate 3000 UHPLC system (Thermo Scientific Dionex, Waltham, MA, USA) with a YMC-Pack ph column (4.6 × 250 mm, 5.0 µm, YMC, Kyoto, Japan), an Inertsil ODS-3 column (4.6 × 250 mm, 5.0 µm, GL Sciences, Kyoto, Japan), and a TSK gel ODS-80Ts column (4.6 × 150 mm, 5.0 µm, TOSOH, Kyoto, Japan). The purity of gentiopicroside, macelignan, γ-mangostin, schisandrol A, schisandrol B, and schisandrin C was determined to be over 95% (Figures S13–S18).

### 2.4. Cell Culture

ER-positive human breast cancer cell line, MCF-7, was obtained from the American Type Culture Collection (ATCC; Manassas, VA, USA). MCF-7 cells were cultured in Roswell Park Memorial Institute-1640 (RPMI-1640) medium (Cellgro, Manassas, VA, USA) and supplemented with 10% fetal bovine serum (Gibco BRL, Grand Island, NY, USA) and an antibiotic solution (100 μg/mL streptomycin and 100 U/mL penicillin) in an incubator gassed with 5% CO_2_ at 37 °C.

### 2.5. E-Screen Assay

The E-screen assay reflects an increase in proliferation rates after treatment of the test substance [21]. In our study, the proliferation rates of MCF cells were measured using the EZ-Cytox assay kit (Daeil Lab Service Co., Seoul, Korea). This kit measures cellular mitochondrial activity upon the conversion of water-soluble tetrazolium salt (WST-1) to insoluble formazan crystals [32,33]. MCF-7 cells were seeded in 24-well plates (1 × 10^5^ cells per well) in a phenol red-free RPMI medium (Gibco BRL, Grand Island, NY, USA) supplemented with an antibiotic solution for 24 h. Charcoal-dextran-stripped human serum at 5% (Innovative Research, Novi, MI, USA) was added to remove estrogen in serum [34,35]. MCF-7 cells were treated with concentrations of 5–100 μM gentiopicroside, macelignan, γ-mangostin, three lignans (schisandrol A, schisandrol B, and schisandrin C), and E2 for 144 h, either with or without 100 nM ICI 182,780 (ICI), an ER antagonist [36,37]. Then, the cells were incubated with Ez-Cytox reagents for 40 min, and the absorbance of the reaction product was measured at 450 nm using a microplate reader (PowerWave XS, Bio Tek Instruments, Winooski, VT, USA).

### 2.6. Western Blot Analysis

MCF-7 cells were seeded in 6-well plates (4 × 10^5^ cells per well) in a phenol red-free RPMI medium (Gibco BRL, Grand Island, NY, USA) supplemented with 5% charcoal-dextran-stripped human serum (Innovative Research, Novi, MI, USA) and an antibiotic solution for 24 h. MCF-7 cells were treated with concentrations of 5–100 μM of schisandrol A and E2 for 24 h, either with or without ICI (100 nM). MCF-7 cells were lysed in an ice-cold radioimmunoprecipitation assay buffer (Cell Signaling Technology, Danvers, MA, USA) with 1 mM phenylmethylsulfonyl fluoride. After quantification using the Pierce™ BCA Protein Assay Kit (Thermo Scientific, Waltham, MA, USA), the total protein (20 µg) from each sample was separated by 10% SDS-PAGE and transferred to a polyvinylidene fluoride (PVDF) membrane. PVDF membranes were incubated overnight with primary antibodies against phospho-extracellular signal-regulated kinase (p-ERK), ERK, phospho-phosphatidylinositol 3-kinase (p-PI3K), PI3K, p-Akt, Akt, p-ERα, ERα, and glyceraldehyde-3-phosphate dehydrogenase (GAPDH) (Cell Signaling Technology). The membranes were then incubated with horseradish peroxidase-conjugated anti-rabbit antibodies (Cell Signaling, Beverly, MA, USA) and visualized on a FUSION Solo Chemiluminescence System (PEQLAB Biotechnologie GmbH, Erlangen, Germany) using ECL Advance Western blotting detection reagents (GE Healthcare, Little Chalfont, UK).

### 2.7. Statistical Analysis

All experiments were performed in triplicate. All analyses were performed using SPSS Statistics ver. 19.0 (SPSS Inc., Chicago, IL, USA). Non-parametric comparisons of samples were conducted using the Kruskal–Wallis test to analyze the results. Differences were considered statistically significant at *p* < 0.05.

## 3. Results

### 3.1. Effects of Compounds on the Proliferation of MCF-7 Cells

**Figure 2:**
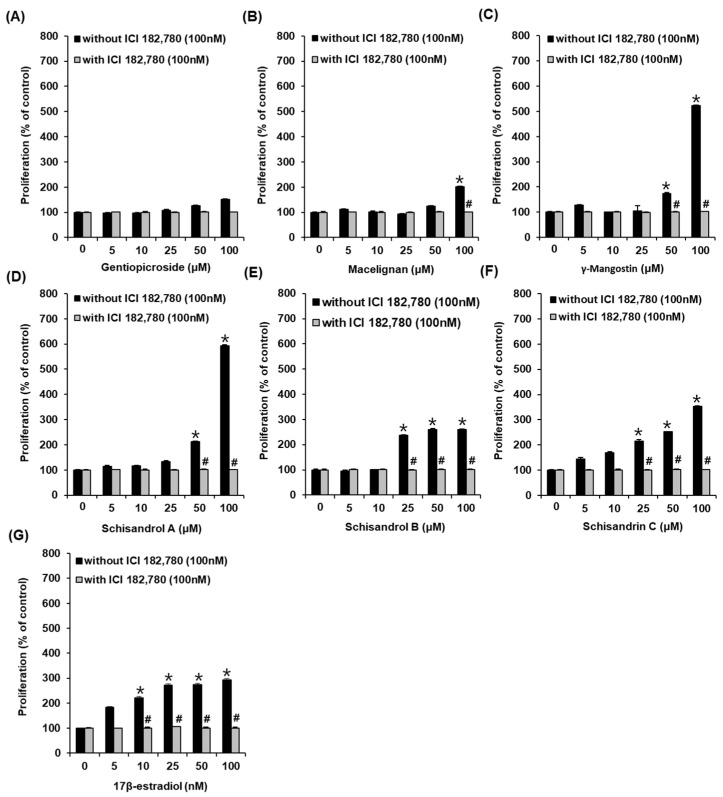
Comparison of estrogenic activities of compounds (**A**–**F**) and (**G**) 17β-estradiol (E2) in the absence or presence of ICI 182,780 (ICI), as determined by cell proliferation measured by E-screen assay in MCF-7 cells. * Significant difference between the cells treated with compounds and the untreated cells. ^#^ Significant reduction by co-treatment with ICI compared to treatment with compounds alone (*n* = 3 independent experiments, *p* < 0.05, Kruskal–Wallis nonparametric test). Data are represented as mean ± SEM.

We examined MCF-7 cell proliferation after treatment with gentiopicroside, macelignan, γ-mangostin, and three lignans (schisandrol A, schisandrol B, and schisandrin C) using Ez-Cytox reagents. Other than gentiopicroside, the compounds promoted cell proliferation in MCF-7 cells (Figure 2A). Cell proliferation increased to 201.07 ± 3.68% after treatment with 100 µM macelignan compared to the untreated cells (Figure 2B), whereas cell proliferation increased to 173.83 ± 4.08% and 522.24 ± 1.94% after treatment with 50 µM and 100 µM γ-mangostin, respectively, compared to the untreated cells (Figure 2C). The proliferation of cells increased to 212.39 ± 1.86% and 592.45 ± 3.73% after treatment with 50 µM and 100 µM schisandrol A, respectively, compared to the untreated cells (Figure 2D). After treatment with 25 µM, 50 µM, and 100 µM schisandrol B, cell proliferation increased to 237.61 ± 0.85%, 259.82 ± 3.91%, and 260.11 ± 3.01%, respectively, compared to the untreated cells (Figure 2E); whereas, after treatment with 25 µM, 50 µM, and 100 µM schisandrin C, cell proliferation increased to 214.91 ± 4.82%, 251.93 ± 1.43% and 235.31 ± 1.72%, respectively, compared to the untreated cells (Figure 2F). Among all the tested compounds, schisandrol A was the most effective in increasing cell proliferation. This effect was mitigated by ICI. Cell proliferation increased to 220.22 ± 4.15%, 272.36 ± 4.86%, 274.64 ± 4.99%, and 292.59 ± 4.22% after treatment with 10 nM, 25 nM, 50 nM, and 100 nM E2, respectively, compared to the untreated cells (Figure 2G). These results proved that schisandrol A was an effective phytoestrogen with E2-like activity that enhanced the proliferation of ER-positive breast cancer cells.

### 3.2. Effect of Schisandrol A on the Protein Expression of p-PI3K, PI3K, p-Akt, Akt, p-ERα, and ERα

**Figure 3:**
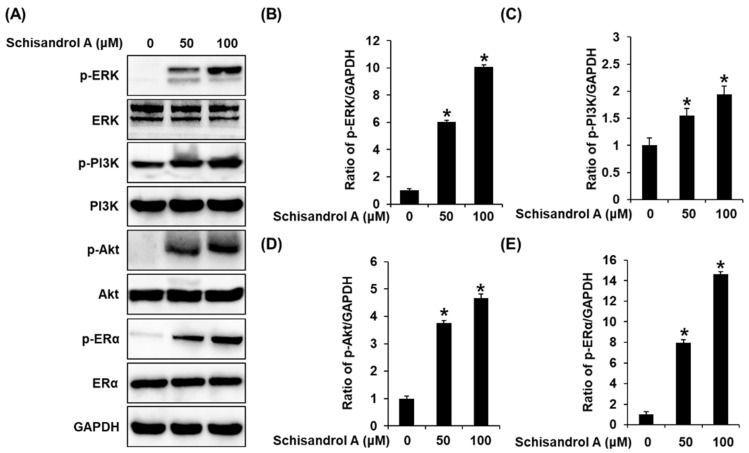
Effect of schisandrol A on the protein expression of phospho-extracellular signal-regulated kinase (p-ERK), ERK, phospho-phosphatidylinositol 3-kinase (p-PI3K), PI3K, p-Akt, Akt, phospho-estrogen receptor α (p-ERα), and ERα in MCF-7 cells: (**A**) protein expression levels of p-PI3K, PI3K, p-Akt, Akt, p-ERα, and Erα and glyceraldehyde 3-phosphate dehydrogenase (GAPDH) in untreated cells and 50 μM and 100 μM schisandrol A-treated MCF-7 cells (24 h). (**B**–**E**) Bar graph representing the densitometric quantification of Western blot bands. * Significant difference between cells treated with schisandrol A and the untreated cells (*n* = 3 independent experiments, *p* < 0.05, Kruskal–Wallis nonparametric test). Data are represented as mean ± SEM.

**Figure 4:**
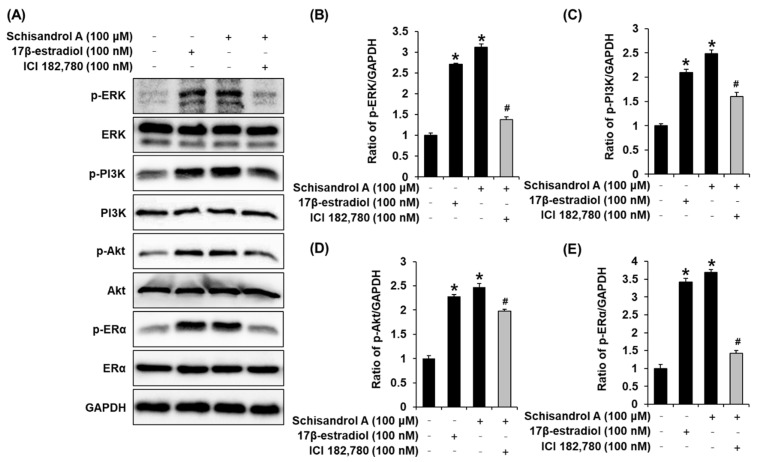
Effect of schisandrol A and 17β-estradiol (E2) on the absence or presence of ICI 182,780 (ICI) in the protein expression of phospho-extracellular signal-regulated kinase (p-ERK), ERK, phospho-phosphatidylinositol 3-kinase (p-PI3K), PI3K, p-Akt, Akt, phospho-estrogen receptor α (p-ERα), and ERα in MCF-7 cells: (**A**) protein expression levels of p-PI3K, PI3K, p-Akt, Akt, p-ERα, and Erα and glyceraldehyde 3-phosphate dehydrogenase (GAPDH) in untreated cells and 50 μM and 100 μM schisandrol A-treated MCF-7 cells (24 h). (**B**–**E**) Bar graph representing the densitometric quantification of Western blot bands. * Significant difference between cells treated with schisandrol A or E2 and the untreated cells. ^#^ Significant reduction in co-treatment with ICI compared to treatment with compounds alone (n = 3 independent experiments, *p* < 0.05, Kruskal–Wallis nonparametric test). Data are represented as mean ± SEM.

To support the proliferation-promoting effects of schisandrol A, the expression of ERα and its related pathways was evaluated using Western blot. Compared with untreated cells, 50 µM and 100 µM schisandrol A induced a concentration-dependent increase in the protein expression of p-ERK, p-PI3K, p-Akt, and ERα (Figure 3). Furthermore, this effect was better than that of 100 µM E2 and was mitigated by treatment with 100 nM ICI. When ICI was present, the expression of p-ERK, p-PI3K, p-Akt, and ERα did not increase after treatment with schisandrol A (Figure 4). These results proved that the responses of ERK, PI3K, and Akt to schisandrol A depend on the functioning of ER.

## 4. Discussion

In previous studies, we reported the estrogenic activity of chemical compounds isolated from plants. Aloe-emodin, rhapontigenin, and chrysophanol 1-*O*-β-d-glucopyranoside were isolated from the roots of *Rheum undulatum* L., sanguiin H-6 was isolated from *Rubus coreanus*, and genistein was isolated from *Pueraria lobata* root [38,39,40]. To detect potential phytoestrogen, in this study, we present evidence supporting the estrogen-like effects of schisandrol A, as well as its possible mechanism of action. Estrogen-like effects were evaluated based on whether MCF-7 proliferation increased after treatment with gentiopicroside, γ-mangostin, and four lignans (macelignan, schisandrol A, schisandrol B, and schisandrin C) in hormone-starved conditions using charcoal-dextran-stripped human serum. Schisandrol A has been reported to have cardioprotective [41], neuroprotective [42,43,44,45], and hepatoprotective effects [46]. Schisandrol B has been demonstrated to possess hepatoprotective [46] and neuroprotective effects [47]. However, its estrogen-like effects remain unclear. Our results show that, except for gentiopicroside, all the compounds promoted MCF-7 cell proliferation, with schisandrol A being the most effective in enhancing cell proliferation. Moreover, this effect was better than that of E2 and was mitigated by the ER antagonist ICI. These results prove that schisandrol A is an effective phytoestrogen with E2-like activity that increases the proliferation of ER-positive breast cancer cells.

The MCF-7 cell model has been extensively used to evaluate the estrogen-like effects of phytoestrogens due to stable estrogen sensitivity and reproducibility [22,48]. Estrogens have been shown to bind and/or activate G protein-coupled ERs (GPERs) [49]. The ERK and PI3K/Akt pathways play important roles in the proliferation of ER-positive breast cancer cells via GPERs [50,51]. As one of the mitogen-activated protein kinase (MAPK) family members, ERK is reported to be associated with cell survival, differentiation, and proliferation [23,24]. Its activation plays an important role in estrogen signaling [52,53,54,55,56]. E2-induced proliferation of MCF-7 cells is associated with the activation of ERK [57]. The E2-induced estrogenic effect was mitigated by treatment with the MEK/ERK inhibitor U0126 [58]. The PI3K/Akt pathway is also an important regulator of ER-positive breast cancer cell proliferation [51,59,60]. Previous studies have reported that treatment with E2 enhances estrogenic activity via the PI3K/Akt pathway, thus increasing the proliferation of ER-positive breast cancer cells [61,62]. The biological effects of estrogen are dependent on the activation of ERα and ERβ. In the nucleus, these receptors act by binding to DNA as ligand-activated transcription factors [63,64]. In addition, previous studies reported that ERα induces cell cycle genes, such as cyclin A2, which lead to cell proliferation and cell cycle stimulation [65,66].

**Figure 5:**
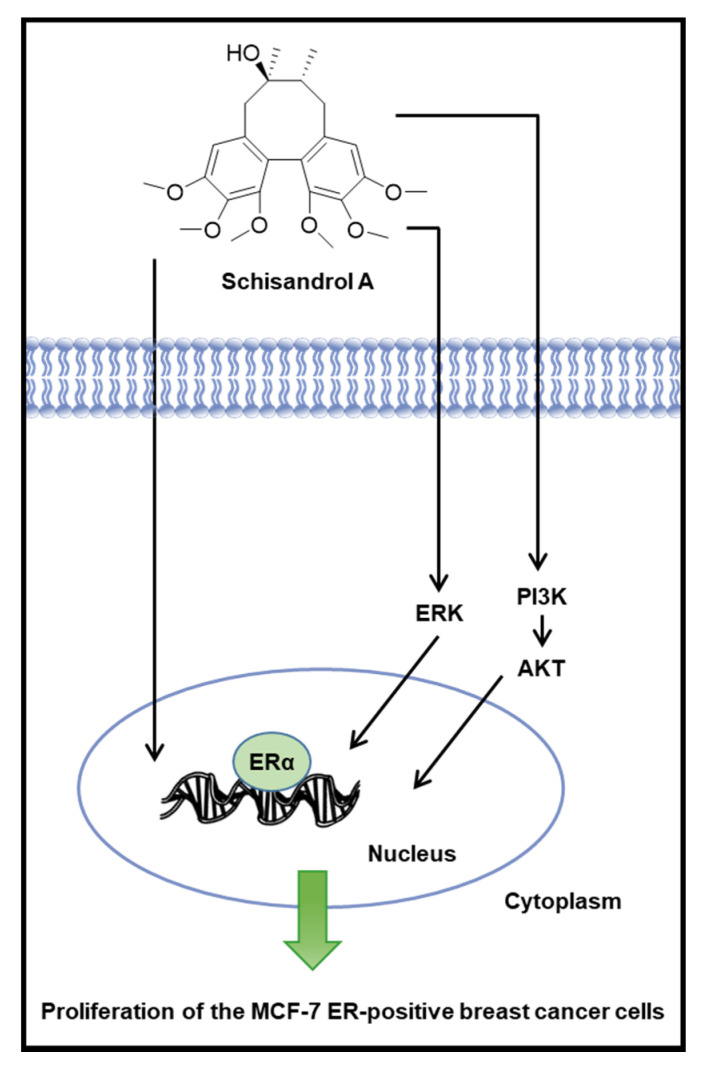
Schematic illustration of the underlying mechanism of the estrogenic activity of schisandrol A via estrogen receptor α (ERα)-dependent signaling pathways in MCF-7 estrogen receptor-positive breast cancer cells.

Our previous study reported the estrogenic activity of sanguiin H-6, which activates the ERα coactivator binding site in MCF-7 cells [39]. In our previous study, genistein exhibited estrogenic activity via the ER pathway in MCF-7 cells [38]. The estrogenic activity of genistein and extract of *Disporum uniflorum* Baker has been reported, and its mechanisms are related to phosphorylation of ERα and ERK [56]. Through progesterone receptor induction and ERα induction via the PI3K/AKT and ERK pathways, the estrogenic activity of black tea and *Dendrobium candidum* extracts has been reported [60]. Our results were consistent with previous studies. It was confirmed that the proliferation-promoting effects of schisandrol A are mediated via the ER-signaling pathway. The treatment with schisandrol A induced a concentration-dependent increase in the protein expression of p-ERK, p-PI3K, p-Akt, and ERα. Another interesting result of the present study is that the effect of schisandrol A on increasing protein expression of p-ERK, p-PI3K, p-Akt, and ERα was better than that of the same concentrations of E2. In addition, when ICI was present, the expression of p-ERK, p-PI3K, p-Akt, and ERα did not increase upon treatment with schisandrol A. ICI binds to ER and downregulates the cellular levels of ER [67,68]. These results prove that the responses of ERK, PI3K, and Akt to schisandrol A depend on the function of normal ER. Taken together, these results indicate that schisandrol A exhibits estrogenic activity via the activation of ERK, PI3K, Akt, and ERα (Figure 5). Although future in-depth studies, including animal experiments with the uterotrophic assays and investigations into the detailed molecular mechanisms are required, this study provides an experimental basis for the application of phytoestrogens.

## 5. Conclusions

In this study, we evaluated the estrogenic effects of gentiopicroside, macelignan, γ-mangostin, and three lignans (schisandrol A, schisandrol B, and schisandrin C). All the three lignans were effective phytoestrogens with proliferation enhancing activity in MCF-7 cells. Among all the compounds, schisandrol A was the most effective in enhancing cell proliferation, and its effect was superior to that of E2. The potential mechanism of action of schisandrol A involves the activation of ERK, PI3K, Akt, and Erα, and it can be used as a chemical constituent to control estrogenic activity.

## Supplementary Materials

The following are available online at https://www.mdpi.com/article/10.3390/pharmaceutics13071082/s1, general experimental procedure, plant material, and extraction and isolation procedures.
