# Supplementary material for: Schisandrol A Exhibits Estrogenic Activity via Estrogen Receptor α-Dependent Signaling Pathway in Estrogen Receptor-Positive Breast Cancer Cells
Source: Pharmaceutics. 2021 Jul 15;13(7):1082. doi: 10.3390/pharmaceutics13071082 (PMC8308983; doi:10.3390/pharmaceutics13071082)
Supplement: Supplementary file 1 [file pharmaceutics-13-01082-s001.zip › pharmaceutics-1270879-supplementary materials/pharmaceutics-1270879-supplementary materials.pdf]

Supporting Information

Extraction and isolation

Gentiana macrophylla (2.0 kg) was extracted with MeOH in the three times. The MeOH extract (427.65 g) was suspended in water and partitioned with *n*-hexane, $CHCl_3$, EtOAc and *n*-BuOH successively, to give the residue of 34.70g of $CHCl_3$ fraction, 14.71 g of EtOAc fraction, *n*-BuOH fraction (160.0 g) and water-soluble fraction. The *n*-BuOH fraction (160.0 g, GMB) was chromatographed over a silica column chromatography using gradient of increasing polarity with $CHCl_3$-MeOH (100:0 to 1:1) as solvents and was fractioned into 12 sub-fractions (GMB1- GMB12). GMB 7 (9.5 g) was subject to RP-MPLC eluted with MeOH-water (0:100 to 80:20) to give 2 sub-fractions (GMB7A- GMB7B). GMB 7B (7.2 g) was purified by silica gel column chromatography by using $CHCl_3$-MeOH (50:1 to 1:1) as solvent and washing by MeOH to give 4 sub fractions (GMB7B1- GMB7B4), including **Gentiopicroside.** The structure was identified by ^{1}H NMR (Fig. S1) and ^{13}C NMR (Fig. S2).

Gentiopicroside: ^{1}H NMR (CD_3OD, 400 MHz) δ 7.46 (1, s, H-3), 5.75 (1H, m, H-8), 5.66 (1H, d, *J* = 2.8 Hz, H-10a), 5.60 (1H, brs, H-1), 5.21(1H, m, H-10b), 5.08 (1H, d, *J* = 17.7 Hz, H-7a), 4.98 (1H, dd, *J* = 17.7, 3.2 Hz, H-7b), 4.65 (1H, d, *J* = 7.9 Hz, H-1′), 3.29 (2H, m, H-9), 3.89 (1H, dd, J = 11.8, 1.6 Hz, H-6′), 3.64 (1H, dd, J = 11.9, 6.2 Hz, H-6′), 3.14 (1H, brt, J= 8.3 Hz, H-2′), 3.30 (2H, m, H-3′,5′); ^{13}C NMR (CD_3OD, 100 MHz) δ 98.5 (C-1), 150.6 (C-3), 104.9 (C-4), 127.0 (C-5), 118.5 (C-6), 71.5 (C-7), 135.0 (C-8), 46.6 (C-9), 117.2 (C-10), 166.3 (C-11), 100.2 (C-1'), 74.5 (C-2′), 78.4 (C-3′), 70.9 (C-4′), 77.9 (C-5′), 62.8 (C-6′).

Reference

1. Hajimehdipoor H, Dijoux-Franca M.G, Mariotte A.M, Amanzadeh Y, Sadat-Ebrahimi S.E, Ghazi-Khansari M. and Mozaffarian V. Phytochemical study of *Swertia longifolia*. *Daru Journal of Pharmaceutical Science*, **2008**, 16(4), 245-249.

2. Ai Mei Yang, Hui Li, Jie Li Liu, Han Han and Jing Sun.; Chemical constituents of *Gentiana algida*. *Chemistry of Natural Compounds*, **2013**, 49(4), 755-756.

3. Bao Chen, Yinghua Peng, Xinhui Wang, Zhiman Li and Yinsi Sun.; Preparative

Separation and Purification of Four Glycosides from Gentianae Radix by High-Speed Conter-Currents Chromatography and Comparison of Their Anti-NO Production Efffects. *Molecules*, **2017**, 22(11), 2002.

Myristical fragrams seeds (600.0 g) was extracted with MeOH in the three times. The MeOH extract (114.38 g) was supended water and partitioned between EtOAc successively, to give the residue EtOAc fraction (98.05 g, MFE). The EtOAc fraction (96.78 g, MFE) was chromatographed over a silica gel colum using gradient with *n*-hexane-EtOAc (100:0 to 1:1) into 5 sub fraction (MFE1-MFE5). MFE3 (34.5 g) was subject to RP-MPLC eluted with MeOH-water (50:5 to 90:10). **Macelignan** was isolated as a precipitate in fraction MFE3C. The structure was identified by ^{1}H NMR (Fig. S3) and ^{13}C NMR (Fig. S4).

Maceliganan: ^{1}H NMR (CD_3OD, 400 MHz) δ 6.81 (1H, d, *J* = 7.9Hz, H-5), 6.71 (1H, d, *J* = 7.9 Hz, H-5′), 6.63 (1H, dd, *J* = 13.4, 1.5 Hz, H-2), 6.61 (1H, dd, *J* = 14.3, 1.9 Hz, H-2′), 5.91 (2H, q, *J* = 1.5 Hz, -OCH_2O-), 3.85 (3H, s, -OCH_3), 2.71(2H, dd, J = 13.7, 5.0 Hz, H-7), 2.26 (2H, mH-7′), 1.72 (2H, m, H-8, 8′), 0.82 (3H, d, *J* = 4.5 Hz, H-9), 0.82 (3H, d, *J* = 4.5 Hz, H-9′); ^{13}C NMR (CD_3OD, 100 MHz) δ 147.6 (C-3), 146.4 (C-3′), 145.6 (C-4), 143.6 (C-4′), 135.8 (C-1), 133.8 (C-1′), 121.9 (C-6), 121.8 (C-6′), 114.1 (C-5′), 111.5 (C-2′), 109.4 (C-5), 108.0 (C-2), 100.8 (-OCH_2O-), 55.9 (-OCH_3), 39.5 (C-8), 39.4 (C-8′), 39.2 (C-7), 38.9 (C-7′), 16.3 (C-9), 16.2 (C-9′).

Reference; Jae Pil Lee, Myung-Gyun Kang, Joon Yeop Lee, Jong Min Oh, Seung Cheol Baek, Hyun Hee Leem, Daeui Park, Myoung-Lae Cho and Hoon Kim. Potent innibition of acetylcholinestresase by sargachromanol I from *Sargassum siliquastrum* and by selected natural compounds. *Bioorganic Chemistry*, **2019**, 89, 103043.

The dried pericarp of *Garcinia mangostanta* L (1.23 kg) was extracted with EtOH in the three times. The EtOH extract (87.1g) was suspended in water and solvent partitioned with $CHCl_3$, EtOAc and *n*-BuOH yielding 50.18 g, 2.74 g, 18.27 g of residue, respectively. The $CHCl_3$ fraction (45.93 g, GMC) was chromatographed over a silica gel column chromatography, using gradient with $CHCl_3$-MeOH (100:0 to 0:100)

as solvent and gave 18 sub-fractions (GMC1-GMC18). GMC12 (4.73 g) fraction was separated using preparative RP-MPLC with 40% -80% MeOH to yield **γ-mangostin**. The structure was identified by ^{1}H NMR (Fig. S5) and ^{13}C NMR (Fig. S6).

γ-mangostin: ^{1}H NMR (CD_3OD, 400 MHz) δ 6.23 (1H, s, H-4), 6.67 (1H, s, H-5), 3.20 (2H, m, H-1′), 5.26 (2H, t, J = 5.4 Hz, H-2′), 1.79 (3H, s, H-4′), 1.66 (3H, s, H-5′), 4.11 (2H, d, J = 5.9 Hz, H-1″), 5.26 (2H, t, J = 5.4 Hz, H-2″), 1.84 (3H, s, H-4″), 1.66 (3H, s, H-5″); ^{13}C NMR (CD_3OD, 100 MHz) δ 160.1 (C-1), 109.7 (C-2), 161.9 (C-3), 91.5 (C-4), 152.6 (C-4a), 99.5 (C-5), 154.8 (C-6), 140.6 (C-7), 128.1 (C-8), 110.8 (C-8a), 182.1 (C-9), 102.5 (C-9a), 151.7 (C-10a), 20.8 (C-1'), 122.5 (C-2′), 130.1 (C-3′), 16.9 (C-4′), 24.6 (C-5′), 25.2 (C-1″), 123.4 (C-2″), 130.2 (C-3″), 16.5 (C-4″), 24.7 (C-5″).

Reference

Xiao-Qian Chi, Cheng-Ting Zi, Hong-Mei Li, Liy Yang, Young-Feng Lv, Jin-Yu Li, Bo Hou, Fu-Cai Ren, Jiang-Miao Hu and Jun Zhou. Design, synthesis and structure activity relationships of mangositn analogs as cytotoxic agents. *RSC Advances*, 72, **2018**, 8(72), 41377-41388.

Dried *Schisandra chinensis* fruits (7.0 kg) were soaked at room temperature for 24 h, extracted three times with MeOH. The MeOH extract (2.56 kg) was suspended in water and partitioned with *n*-hexane, $CHCl_3$, EtOAc, and *n*-BuOH successively, to produce a *n*-hexane extract (180.25 g), a $CHCl_3$ extract (52.73 g), an EtOAc extract (205.87 g), a *n*-BuOH extract (809.88 g), and a water extract (1307.13 g), respectively. The crude *n*-hexane extract (170.09 g, SCH) was applied to a silica column chromatography using gradients of increasing polarity with *n*-hexane-EtOAc (100:0 to 2:1) and $CHCl_3$-MeOH (20:1 to 1:1) as solvents, this being fractionated into 18 sub-fractions (SCH1-SCH18). **Schisanrin C** was precipitated from fraction SCH5 in MeOH. SCH6 (14.3 g) was fractionated using a RP-MPLC with MeOH-water (5:95 to 100:0) into 23 sub fractions (SCH6A-SCH6W). In fraction SCH6V in MeOH, **Schisandrol B** was precipitated.

Fractions SCH11, SCH12, and SCH-13 (SCH13, 22.6 g) were combined and applied to a silica column chromatography using gradients of increasing polarity with *n*-hexane-EtOAc (30:1 to 2:1) and $CHCl_3$-MeOH (20:1 to 1:1) as solvents, with these being fractionated into 11 sub fractions (SCH13A-SCH13K). Fractions SCH13I and SCH13J (SCH13J, 13.2 g) were combined and separated using a RP-MPLC with MeOH-H_2O (30:70 to 100:0) to give 10 sub-fractions (SCH13J1-SCH13J10); **Schisandrol A** was isolated as a precipitate in fraction SCH-13J4. All compounds were identified by ^{1}H NMR (Fig. S7, S9, S11) and ^{13}C NMR (Fig. S8, S10, S12).

Reference

Pisey Pel, Hee-Sung Chae, Piseth Nhoek, Woojin Yeo, Young-Mi Kim, Young-Won Chin., Lignans from the fruits of *Schisandra chinensis* (Turcz.) Baill inhibit proprotein convertase subtilisin/kexin type 9 expression., *Phytochemistry,* **2017**, 136, 116-124.

Schisandrol A: ^{1}H NMR ($CDCl_3$, 400 MHz) δ 6.62 (1H, s, H-4), 6.55 (1H, s, H-11), 3.90(12H, m, -OCH_3 ×4), 3.59 (6H, -OCH_3 ×2), 2.67 (2H, m, H-6α, 9β), 2.39 (2H, m, H-6β, 9α), 1.89 (1H, m, H-8), 1.27 (3H, s, H-7), 0.83 (3H, d, J = 7.1 Hz, H-8); ^{13}C NMR ($CDCl_3$, 100 MHz) δ 152.3 (C-12), 152.0 (C-14), 151.8 (C-3), 151.5 (C-1), 140.7 (C-2), 140.1 (C-5), 133.9 (C-13), 131.8 (C-10), 124.1 (C-16), 122.7 (C-15), 110.4 (C-4), 109.9 (C-11), 71.8 (C-8), 61.0 (-OCH_3 ×2), 60.7 (-OCH_3), 60.6 (-OCH_3), 56.0 (-OCH_3), 55.9 (-OCH_3), 41.8 (C-6), 40.8 (C-9), 34.2 (C-7), 29.9 (C-18), 15.9 (C-17).

Reference

1. Yongbei Liu, Yupei Yang, Shumaila Tasneem, Nusrat Hussain, Muhammad Daniyal, Hanwen Yuan, Qingling Xie, Bin Liu, Jing Sun, Yuqing Jian, Bin Li, shenghuang Chen and Wei Wang. Lignans from Tujia Ethnomedicine heilaohu: Chemical characterizion and Evaluation of Their Cytotoxicity and Antioxidant Activities. *Molecules*, **2018**, 23(9), 2147.

2. Chenning Zhang, Xu Zhao, Xin Mao, Aijing zhi Liu, Xiaolong Li, Kaishun Bi and

Ying Jia. Pharmacological evaluation of sedative and hypnotic effects of schizandrin through the modification of pentobarbital-induced sleep behaviors in mice. *European Journal of Pharmacology*, **2014**, 744, 157-163.

Schisandrol B: ^{1}H NMR ($CDCl_3$, 400 MHz) δ 6.62 (1H, s, H-2), 6.48 (1H, s, H-2′), 5.97 (2H, dd, *J* = 4.9, 1.5 Hz, $-OCH_2O-$), 3.91 (3H, s, $-OCH_3$), 3.91 (3H, s, $-OCH_3$), 3.84 (3H, s, $-OCH_3$), 3.50 (3H, s, $-OCH_3$), 2.69 (1H, d, *J* = 13.5 Hz, H-7a), 2.58 (1H, dd, *J* = 14.1, 1.5 Hz, H-7a′), 2.34 (2H, m, H-7b, 7b′), 1.87 (2H, m, H-8′), 1.26 (3H, s, H-9), 0.82 (3H, d, *J* = 7.3 Hz, H-9′); ^{13}C NMR ($CDCl_3$, 100 MHz) δ 152.3 (C-3), 152.1 (C-5), 147.8 (C-3′), 141.1 (C-5′), 140.7 (C-4), 134.9 (C-4′), 132.4 (C-1′), 132.0 (C-1), 124.1 (C-6), 121.8 (C-6′), 110.2 (C-2), 105.9 (C-2′), 100.8 ($-OCH_2O-$), 71.6 9C-8), 61.0 ($-OCH_3$), 60.6 ($-OCH_3$), 59.7 ($-OCH_3$), 55.9 ($-OCH_3$), 42.0 (C-8′), 40.4 (C-7), 33.6 (C-7′), 30.1 (C-9), 15.8 (C-9′).

Reference

Chi-Keung Wan, Guo-Yuan Zhu, Xiao-Ling Shen, apurba Chattopadhyay, Saival Dey and Wang-Fun Fong. Gomisin alters substrate interaction and reverses P-glycoprotein-mediated multidrug resistance in HepG2-DR cells. *Pharmacology*, **2006**, 72(7), 824-837.

Schisandrin C: ^{1}H NMR ($CDCl_3$, 400 MHz) δ 6.48 (2H, s, H-4, 11), 3.83 (3H, d, *J* = 1.3 Hz, $-OCH_3$), 3.82 (3H, d, *J* = 1.3 Hz, $-OCH_3$), 2.54 (1H, dd, *J* = 13.6, 7.1 Hz, H-6a), 2.44 (1H, brd, *J* = 13.6, H-6b), 2.24 (1H, dd, *J* = 13.0, 9.6 Hz, H-9a), 2.00 (1H, brd, *J* = 13.3 Hz, H-9b), 1.87 (1H, m, H-8), 1.76 (1H, m, H-7), 0.96 (3H, d, *J* = 7.1 Hz, H-18), 0.72 (3H, d, *J* = 7.1 Hz, H-17); ^{13}C NMR ($CDCl_3$, 100 MHz) δ 141.2 (C-1), 134.7 (C-2), 147.6 (C-3), 106.1 (C-4), 132.7 (C-5), 38.8 (C-6), 33.6 (C-7), 40.7 (C-8), 35.3 (C-9), 138.2 (C-10), 103.1 (C-11), 148.6 (C-12), 134.3 (C-13), 141.0 (C-14), 121.0 (C-15), 122.1 (C-16), 12.5 (C-17), 21.7 (C-18) 100.7 ($-OCH_2O-$), 59.6 ($-OCH_3$).

Reference

Shan-Shan Guo, Xue Pang, Yang Wang, Zhu-Feng Geng, Ju-Qin Cao, Jun-Yu Liang, Zhi-Wei Deng and Shu-Shan Du. Chemical constituents isolated from stems of *Schisandra chinensis* and their antifeedant activity against *Tribolium castaneum*. *Natural Products Research*, **2020**, 34(18), 2595-2601.

The UHPLC UV chromatogram for Compounds purity analysis

Chromatographic separation of the analyte for **Gentiopicroside** purity analysis was performed on Ultimate 3000 UHPLC system (Thermo scientific, USA) with YMC-Pack ph column (4.6 × 250 mm, 5.0 μm, YMC, Japan). An aliquot of the sample (10 μL) was injection into the UHPLC system for analysis. The mobile phase consisted of 0.1% formic acid in water (A) and 0.1% formic acid in MeOH (B). The mobile phase consisting of (A) and (B) was transported at a flow rate of 1.0 mL/min by the following programmed gradient elution: 15 % (B, v/v) isocratic for 15.0 min, 15→25 % (B) in 5.0 min, 25→100 % (B) in 3.0 min, 100 % (B) isocratic for 2.0 min, 100→15 % (B) in 1.0 min, 15 % (B) isocratic for 2.0 min as post-run for reconditioning. The column temperature was maintained at 25 ℃ and the wavelength was at 280 nm. (Fig. S13)

Chromatographic separation of the analyte for **Macelignan** purity analysis was performed on Ultimate 3000 UHPLC system (Thermo scientific, USA) with Inertsil ODS-3 column (4.6 × 250 mm, 5.0 μm, GL Sciences, Japan). An aliquot of the sample (5 μL) was injection into the UHPLC system for analysis. The mobile phase consisted of 0.1 % formic acid in water (A), 0.1 % formic acid in MeCN: MeOH (1:1 v/v, B). The mobile phase consisting of (A) and (B) was transported at a flow rate of 1.0 mL/min by the following programmed gradient elution: 86 % (B, v/v) isocratic for 20.0 min, 86→100 % (B) in 5.0 min, 100 % (B) isocratic for 3.0 min, 100→86 % (B) in 1.0 min, 86 % (B) isocratic for 1.0 min as post-run for reconditioning. The column temperature was maintained at 25 ℃ and the wavelength was at 280 nm. (Fig. S14)

Chromatographic separation of the analyte for **γ-mangostin** purity analysis was performed on Ultimate 3000 UHPLC system (Thermo scientific, USA) system with TSKgel ODS-80Ts column (4.6 × 150mm, 5.0 μm, TOSOH, Japan). An aliquot of the sample (2 μL) was injection into the UHPLC system for analysis. The mobile phase consisted of 0.1 % formic acid in water (A) and 0.1 % formic acid in MeOH (B). The mobile phase consisting of (A) and (B) was transported at a flow rate of 1.0 mL/min by the following programmed gradient elution: 70 % (B, v/v) isocratic for 20.0 min, 70→100 % (B) in 3.0 min, 100 % (B) isocratic for 3.0 min, 100→70 % (B) in 1.0 min, 70 % (B) isocratic for 1.0 min as post-run for reconditioning. The column temperature

was maintained at 25 ℃ and the wavelength was at 270 nm. (Fig. S15)

Chromatographic separation of the analyte for **Schisandrol A, B** and **Schisandrin C** purity analysis were performed on Ultimate 3000 UHPLC system (Thermo scientific, USA) system with Inertsil ODS-3 column (4.6 × 250mm, 5.0 μm, GL Sciences, Japan). An aliquot of the sample (5 μL) was injection into the UHPLC system for analysis. The mobile phase consisted of 0.1 % formic acid in water (A) and 0.1 % formic acid in MeOH (B). The mobile phase consisting of (A) and (B) was transported at a flow rate of 1.0 mL/min by the following programmed gradient elution: 70 % (B, v/v) isocratic for 8.0 min, 70→80 % (B) in 4.0 min, 80→90 % (B) in 8.0 min, 90 % (B, v/v) isocratic for 10.0 min, 90→100 % (B) in 2.0 min, 100 % (B) isocratic for 3.0 min, 100→70 % (B) in 1.0 min, 70 % (B) isocratic for 4.0 min as post-run for reconditioning. The column temperature was maintained at 25 ℃ and the wavelength was at 254 nm. (Fig. S16, S17, S18)

Contents

Figure S1. The ^{1}H NMR spectrum of Gentiopicroside (CD_3OD, 400 MHz)

Figure S2. The ^{13}C NMR spectrum of Gentiopicroside (CD_3OD, 100 MHz)

Figure S3. The ^{1}H NMR spectrum of Macelignan ($CDCl_3$, 400 MHz)

Figure S4. The ^{13}C NMR spectrum of Macelignan ($CDCl_3$, 100 MHz)

Figure S5. The ^{1}H NMR spectrum of γ-mangostin (CD_3OD, 400 MHz)

Figure S6. The ^{13}C NMR spectrum of γ –mangostin (CD_3OD, 100 MHz)

Figure S7. The ^{1}H NMR spectrum of Schisandrol A ($CDCl_3$, 400 MHz)

Figure S8. The ^{13}C NMR spectrum of Schisandrol A ($CDCl_3$, 100 MHz)

Figure S9. The ^{1}H NMR spectrum of Schisandrol B ($CDCl_3$, 400 MHz)

Figure S10. The ^{13}C NMR spectrum of Schisandrol B ($CDCl_3$, 100 MHz)

Figure S11. The ^{1}H NMR spectrum of Schisandrin C ($CDCl_3$, 400 MHz)

Figure S12. The ^{13}C NMR spectrum of Schisandrin C ($CDCl_3$, 400 MHz)

Figure S13. The UHPLC UV chromatogram of Gentiopicroside (280 nm)

Figure S14. The UHPLC UV chromatogram of Macelignan (280 nm)

Figure S15. The UHPLC UV chromatogram of γ –mangostin (270 nm)

Figure S16. The UHPLC UV chromatogram of Schisandrol A (254 nm)

Figure S17. The UHPLC UV chromatogram of Schisandrol B (254 nm)

Figure S18. The UHPLC UV chromatogram of Schisandrin C (254 nm)

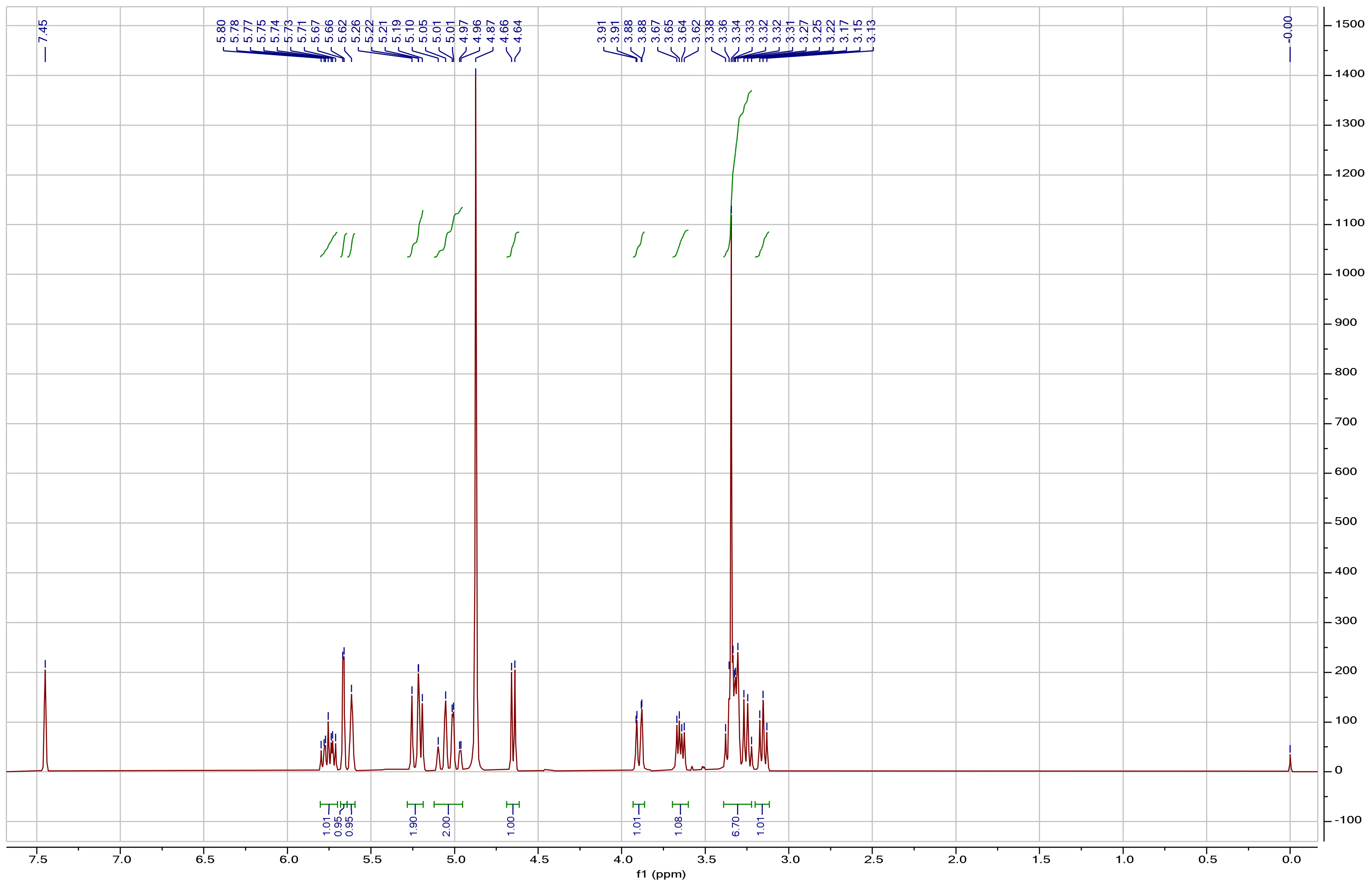

Figure S1. The ^{1}H NMR spectrum of Gentiopicroside (CD_3OD, 400 MHz)

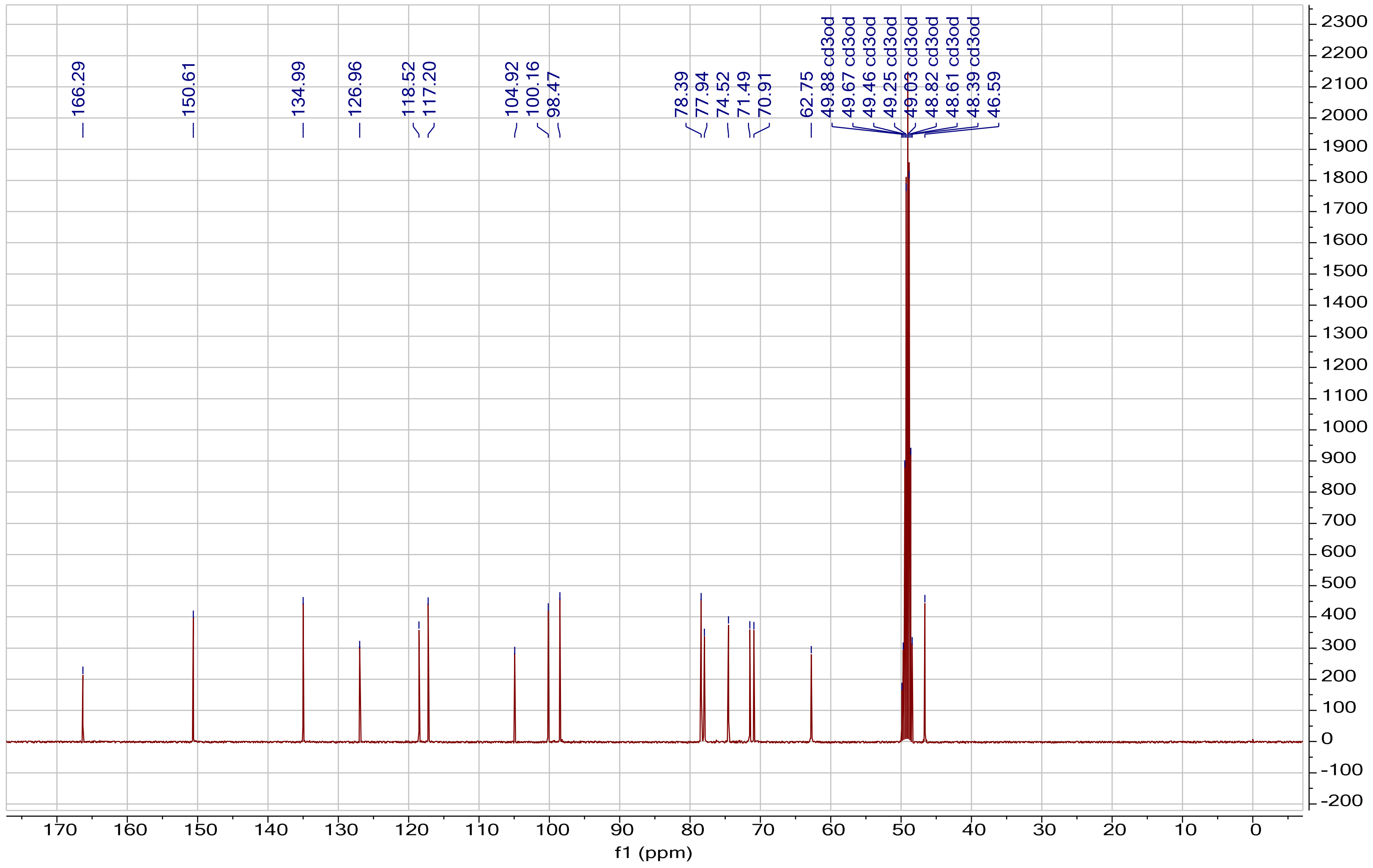

Figure S2. The ^{13}C NMR spectrum of Gentiopicroside (CD_3OD, 100 MHz)

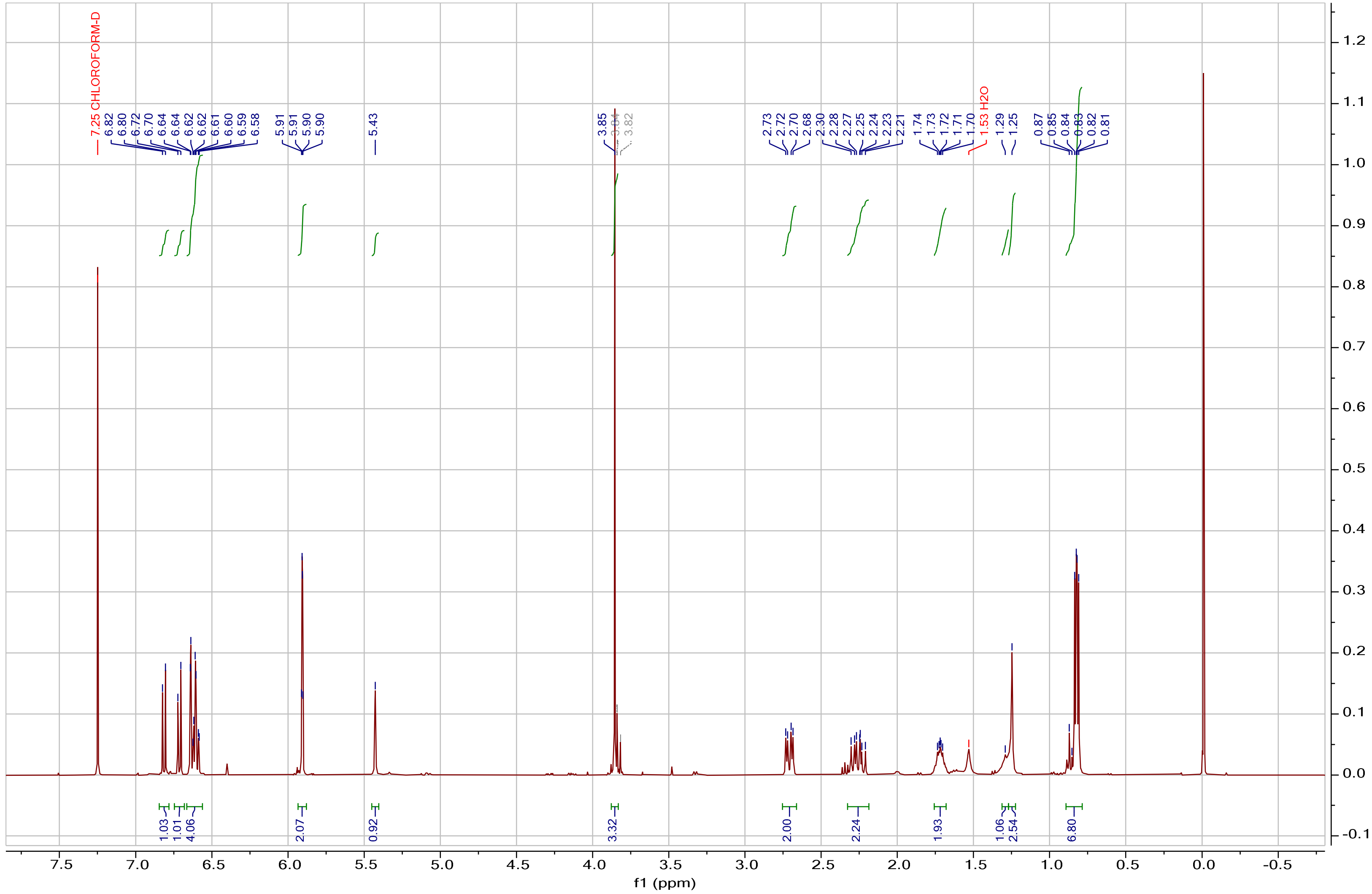

Figure S3. The ^{1}H NMR spectrum of Macelignan ($CDCl_3$, 400 MHz)

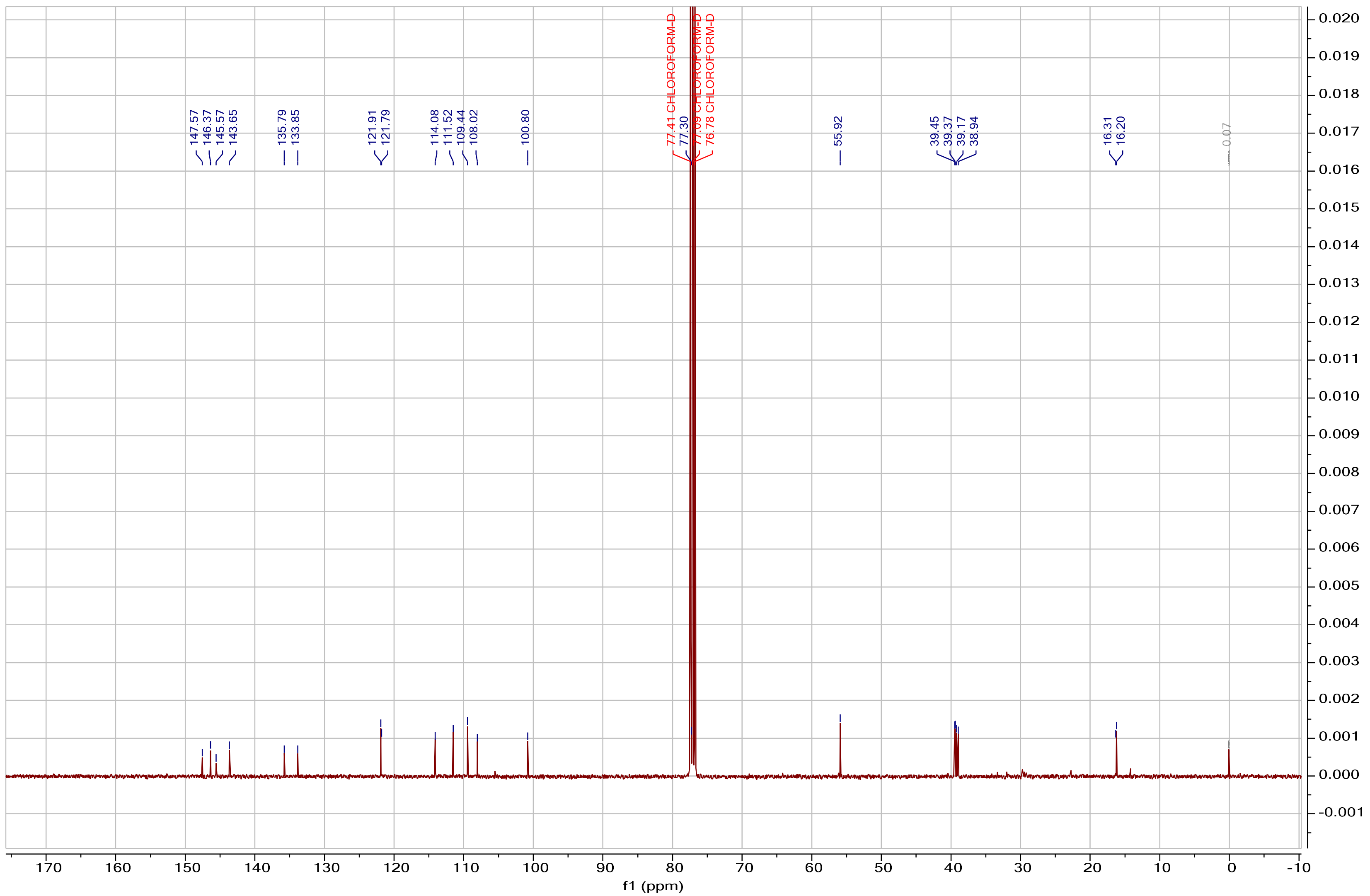

Figure S4. The ^{13}C NMR spectrum of Macelignan ($CDCl_3$, 100 MHz)

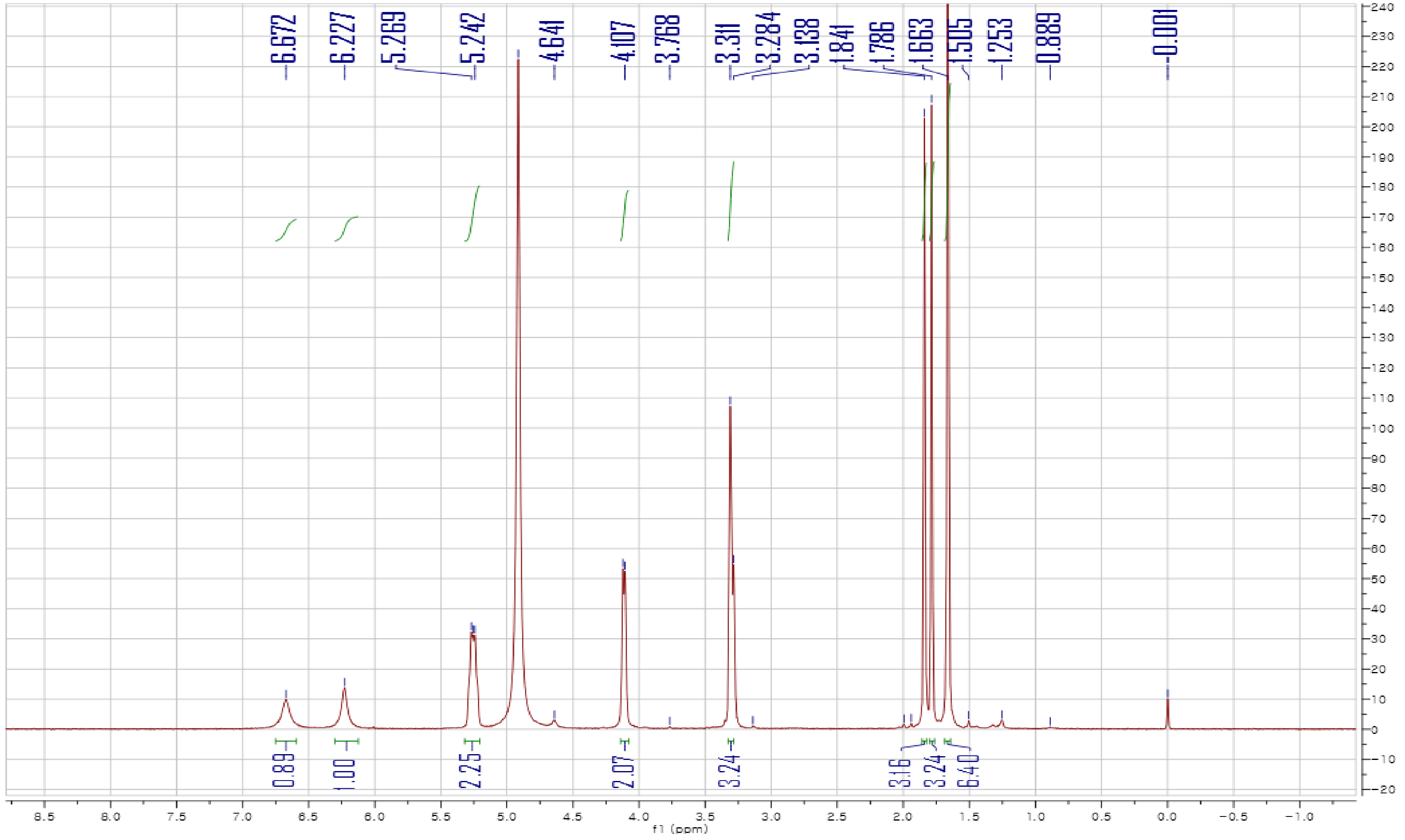

Figure S5. The ^{1}H NMR spectrum of γ-mangostin (CD_3OD, 400 MHz)

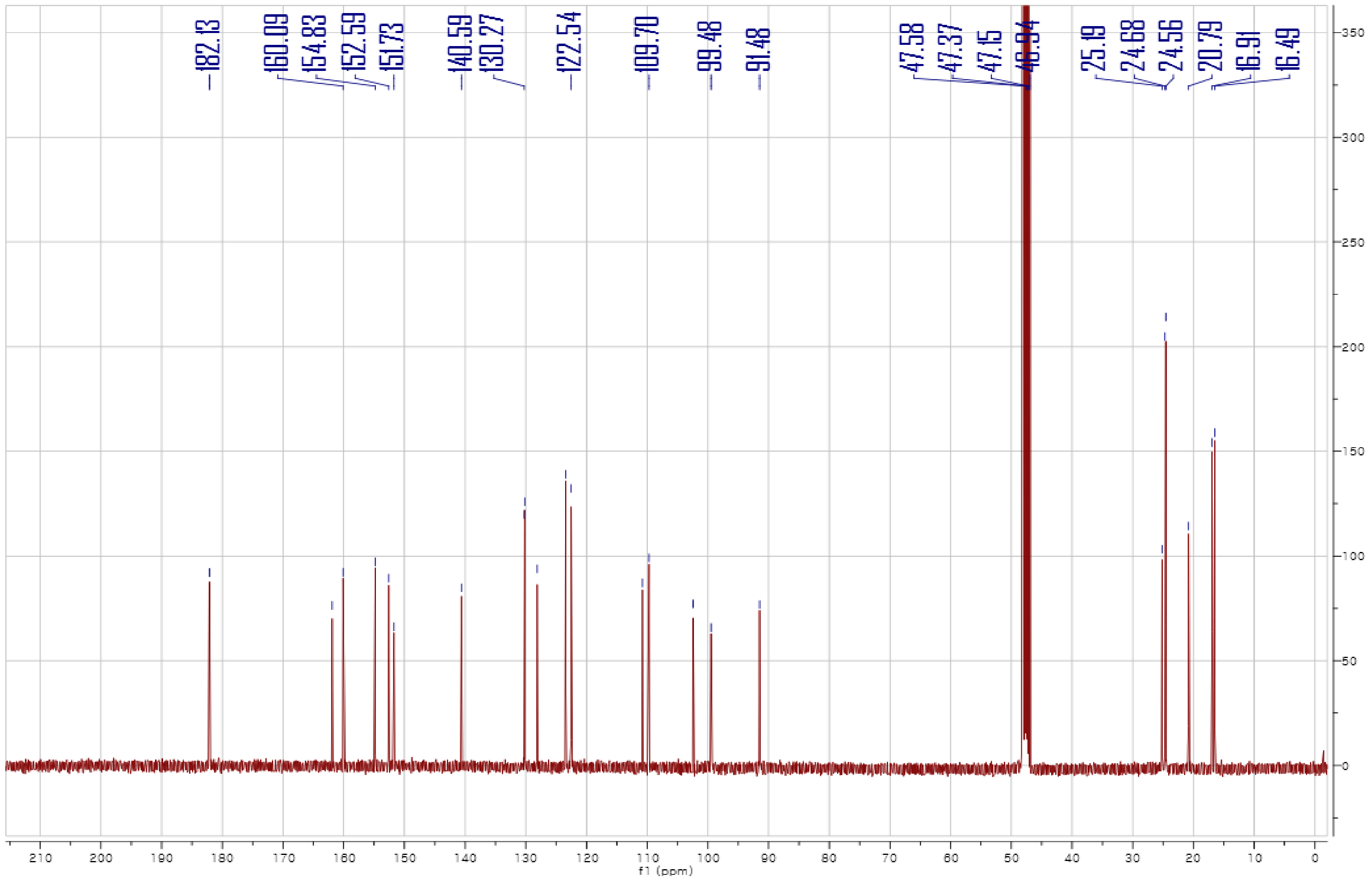

Figure S6. The ^{13}C NMR spectrum of γ -mangostin (CD_3OD, 100 MHz)

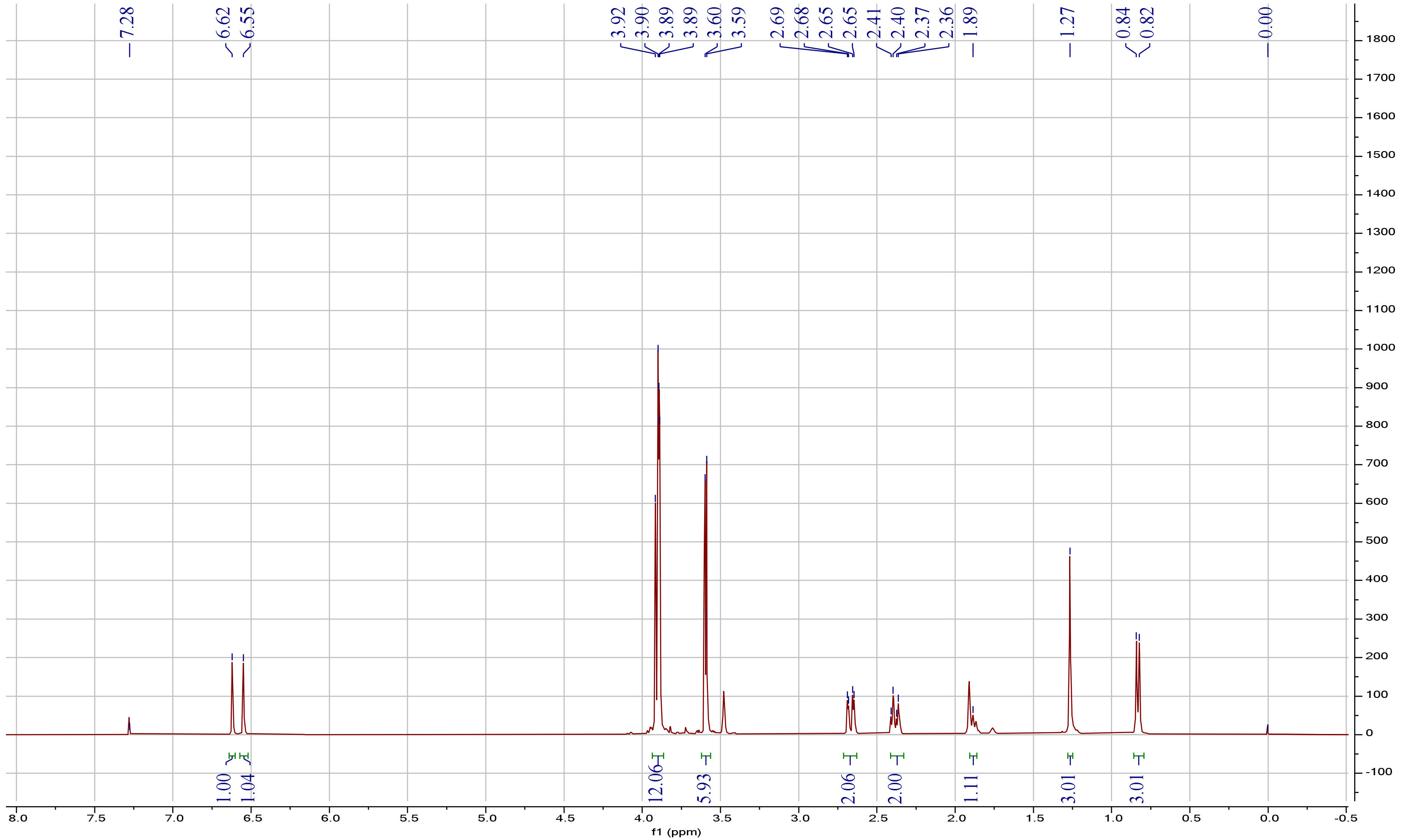

Figure S7. The ^{1}H NMR spectrum of Schisandrol A ($CDCl_3$, 400 MHz)

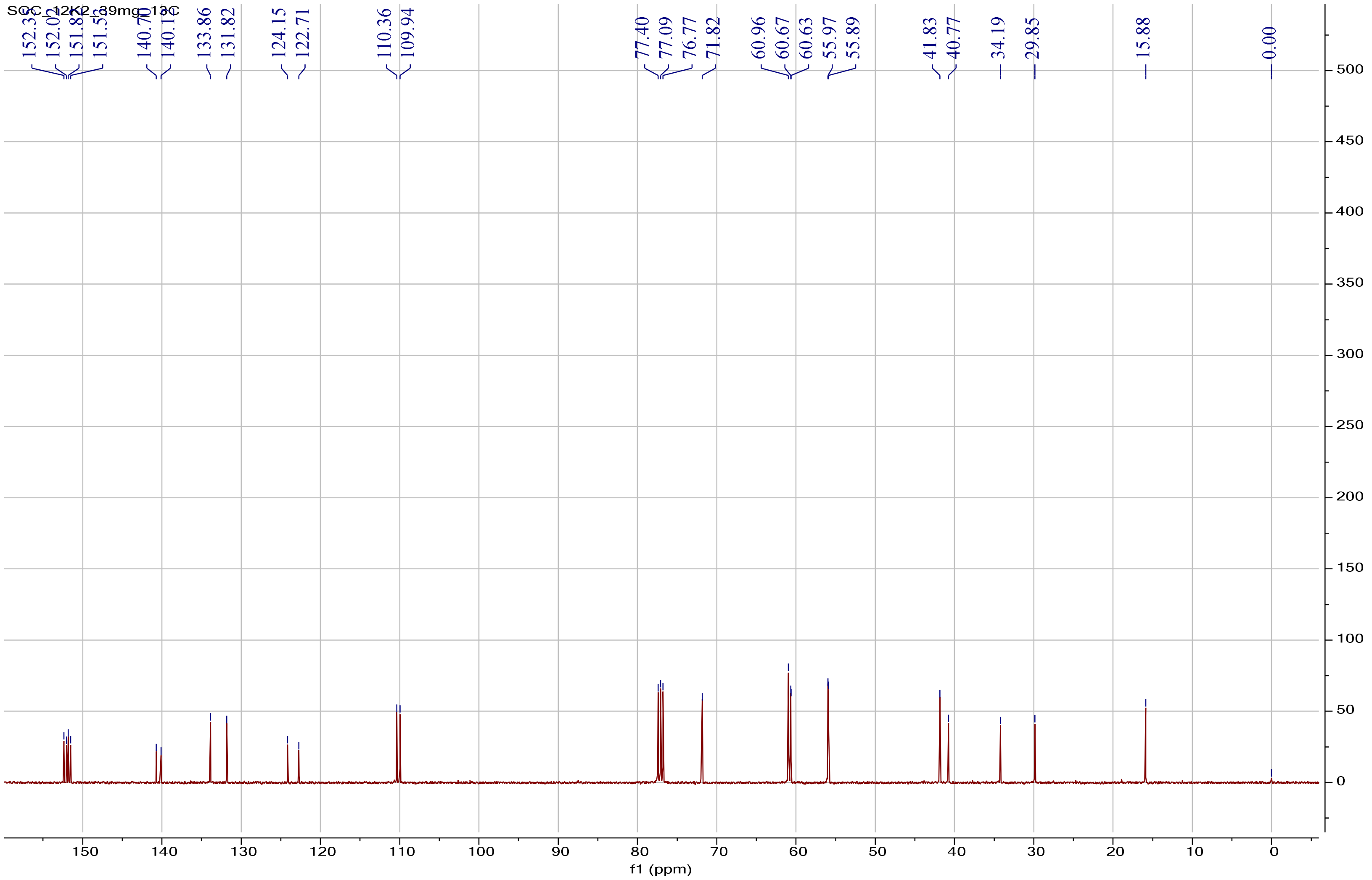

Figure S8. The ^{13}C NMR spectrum of Schisandrol A ($CDCl_3$, 100 MHz)

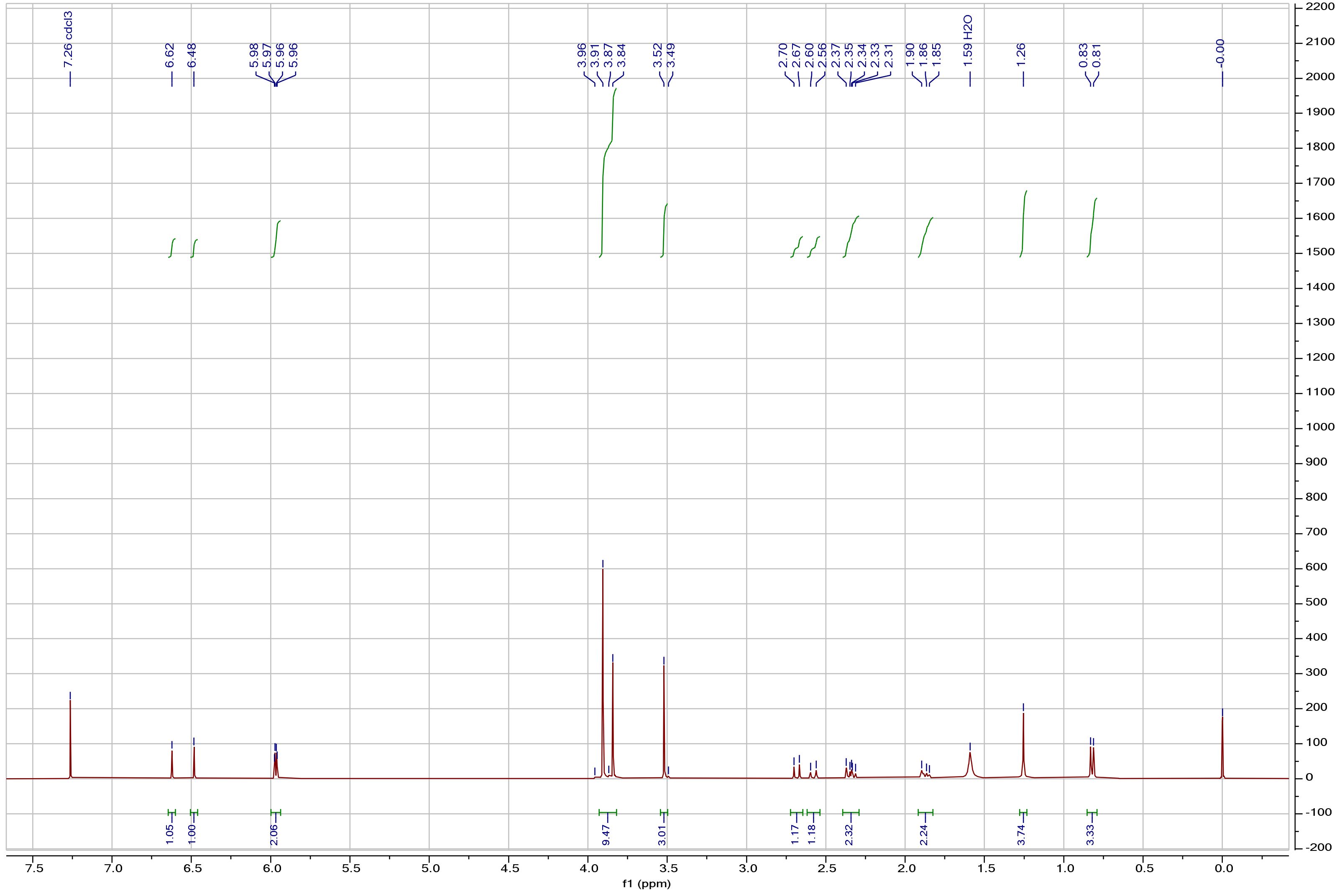

Figure S9. The ^{1}H NMR spectrum of Schisandrol B ($CDCl_3$, 400 MHz)

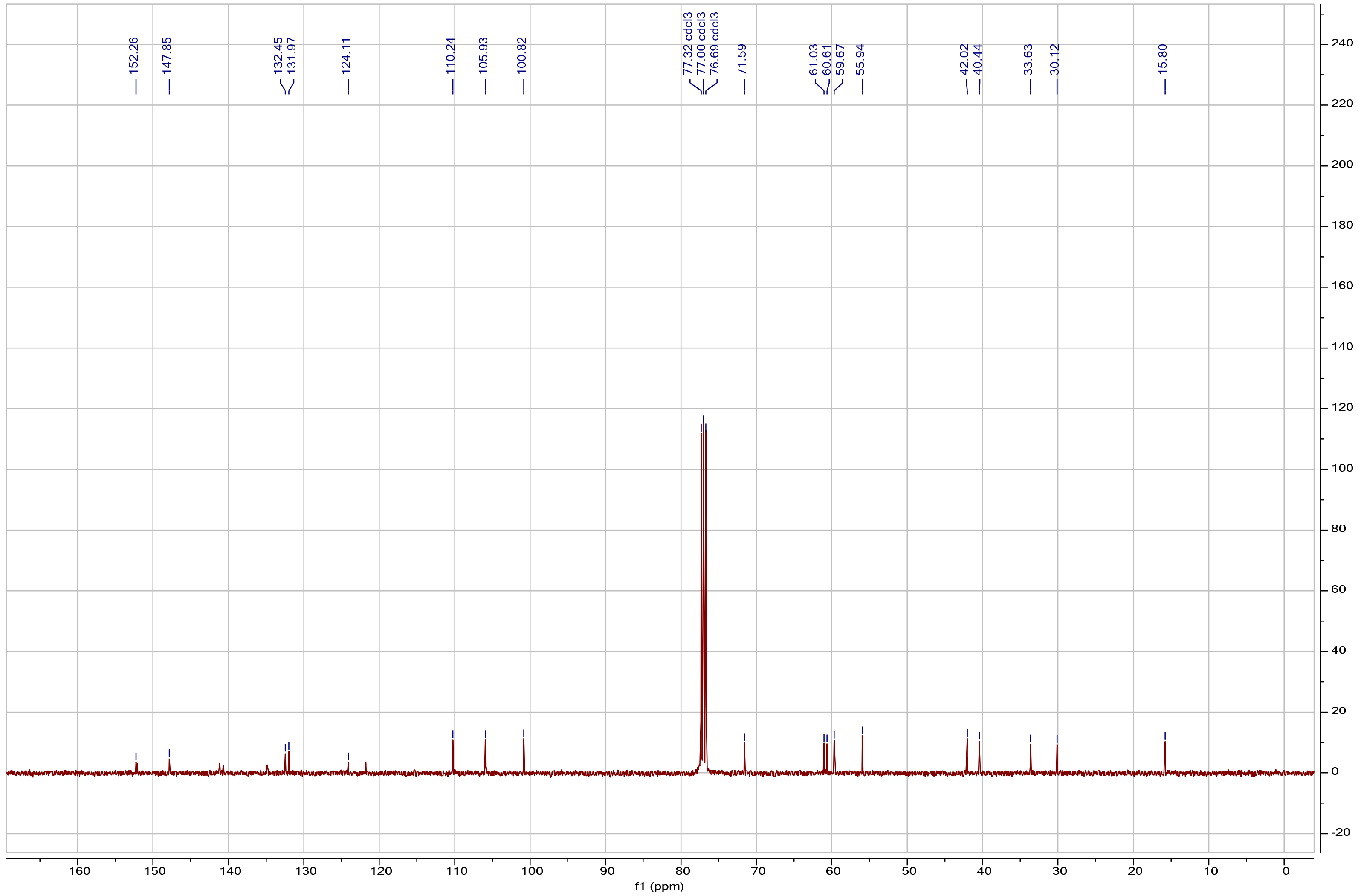

Figure S10. The ^{13}C NMR spectrum of Schisandrol B ($CDCl_3$, 100 MHz)

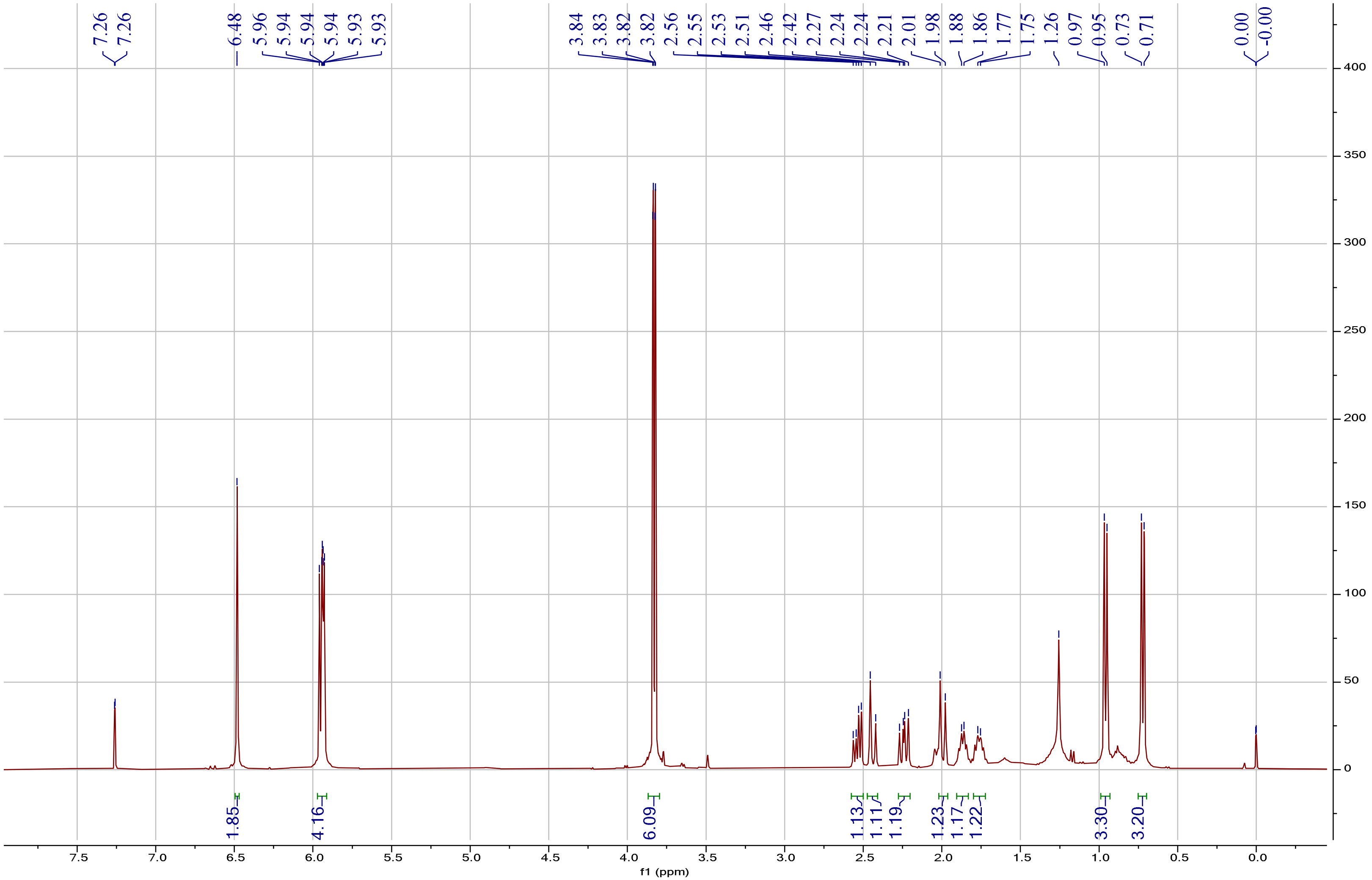

Figure S11. The ^{1}H NMR spectrum of Schisandrin C ($CDCl_3$, 400 MHz)

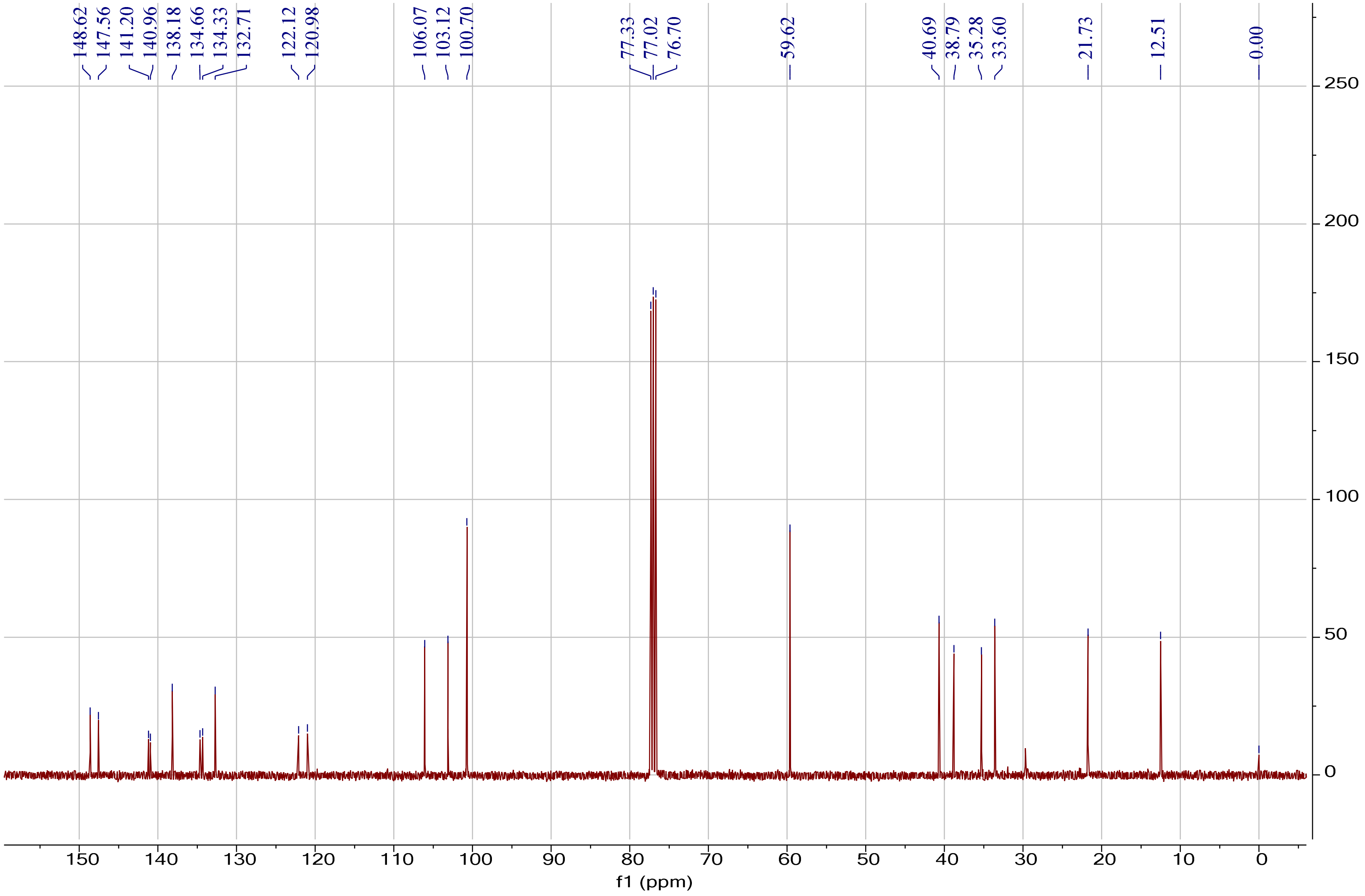

Figure S12. The ^{13}C NMR spectrum of Schisandrin C ($CDCl_3$, 100 MHz)

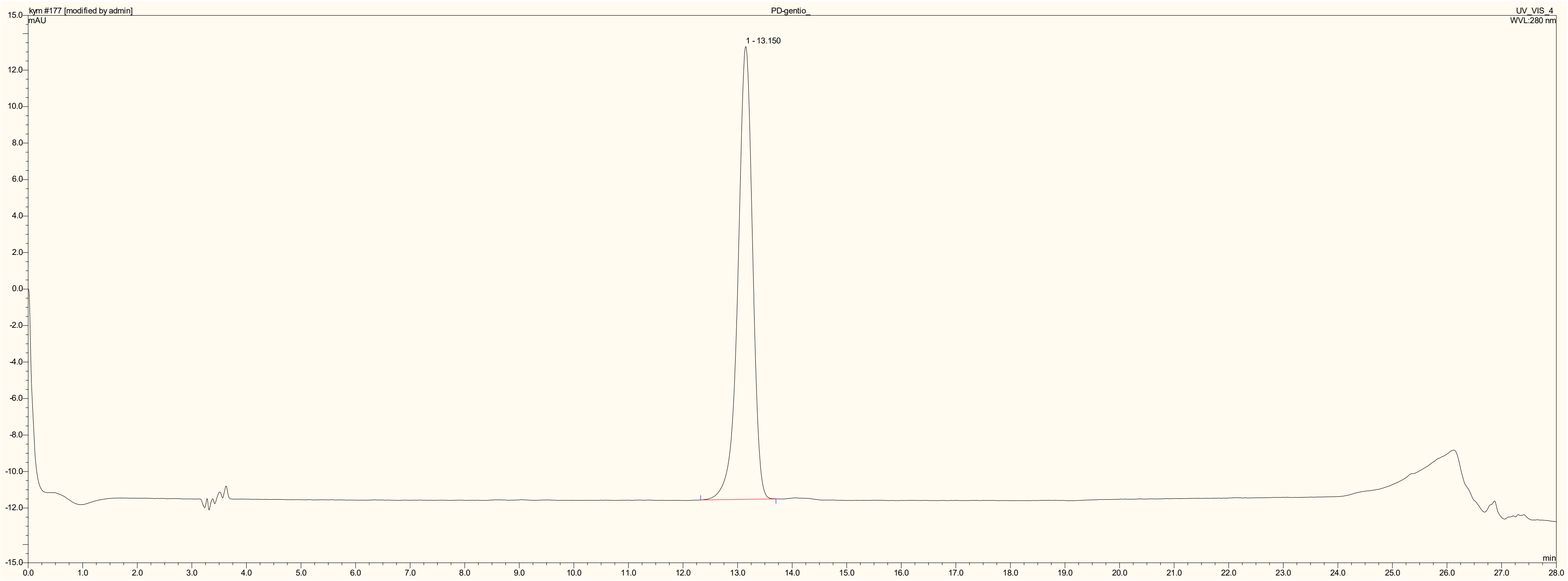

Figure S13. The UHPLC UV chromatogram of Gentiopicroside (280 nm)

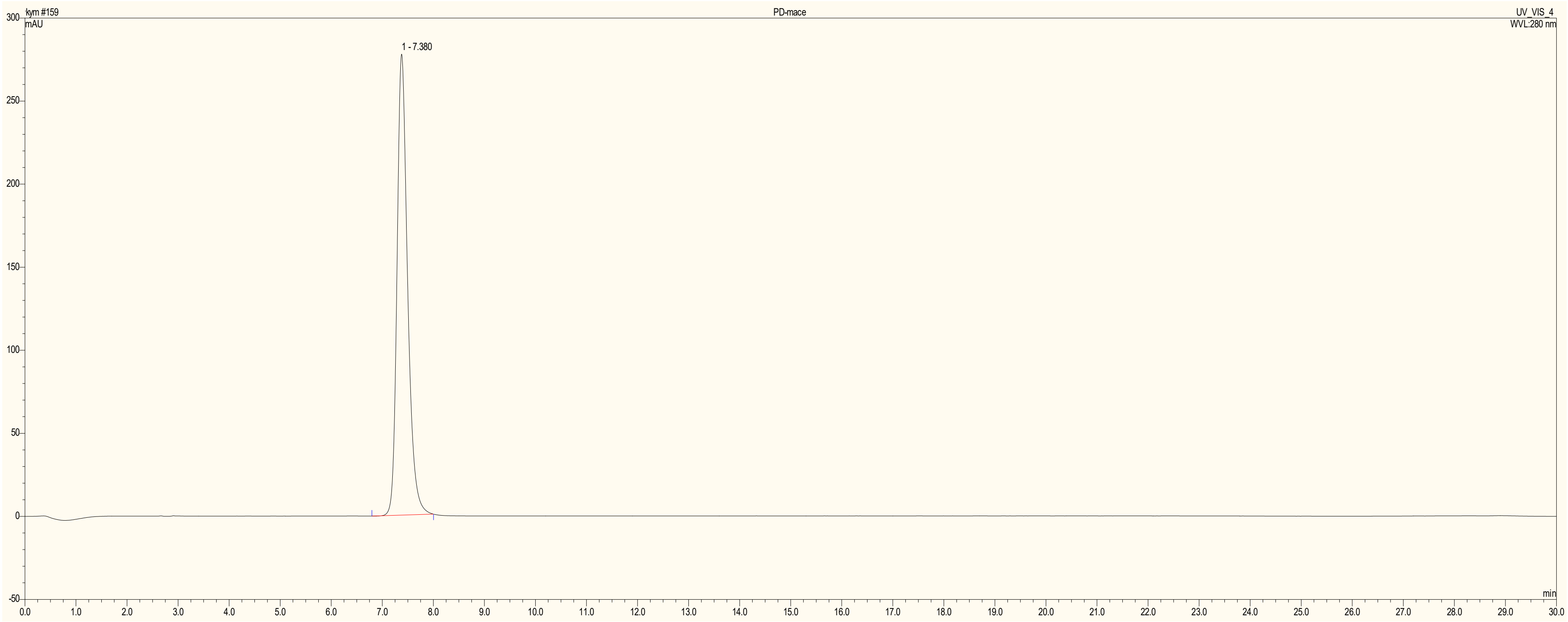

Figure S14. The UHPLC UV chromatogram of Macelignan (280 nm)

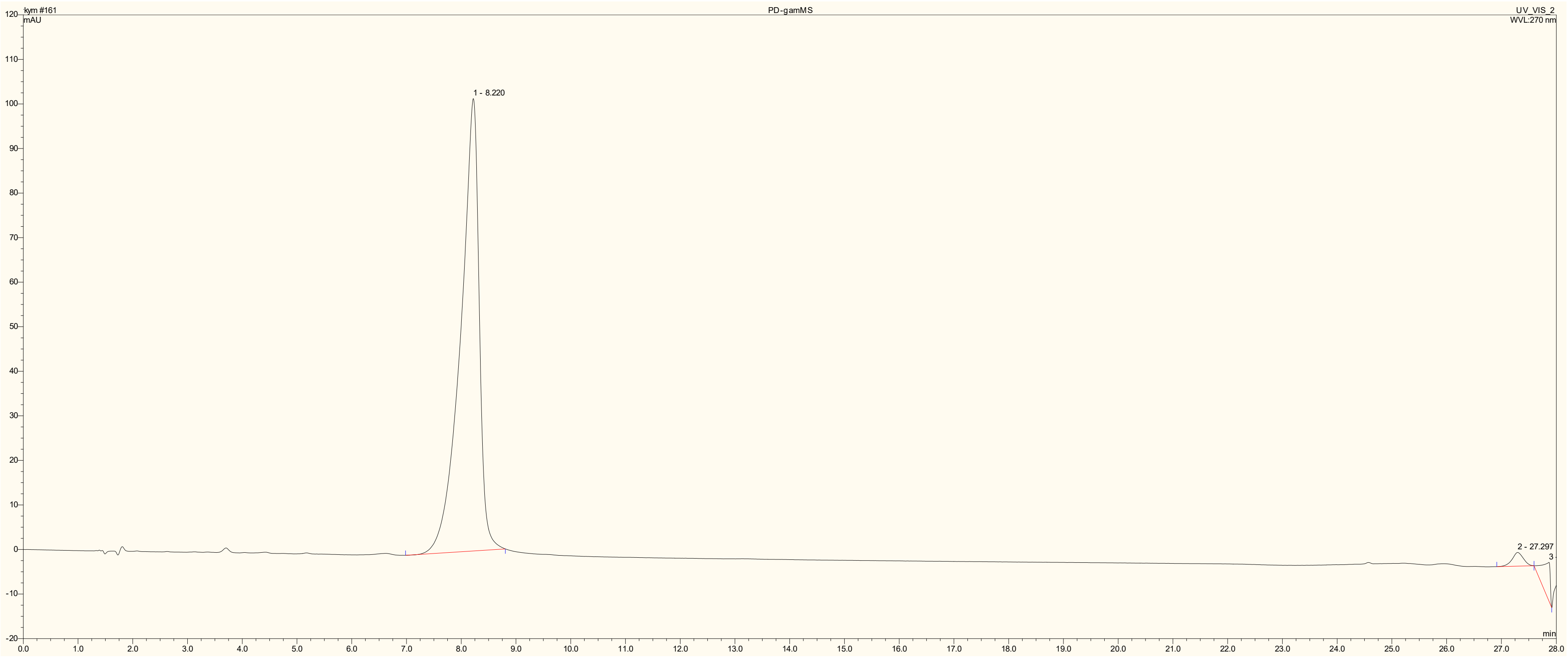

Figure S15. The UHPLC UV chromatogram of γ –mangostin (270 nm)

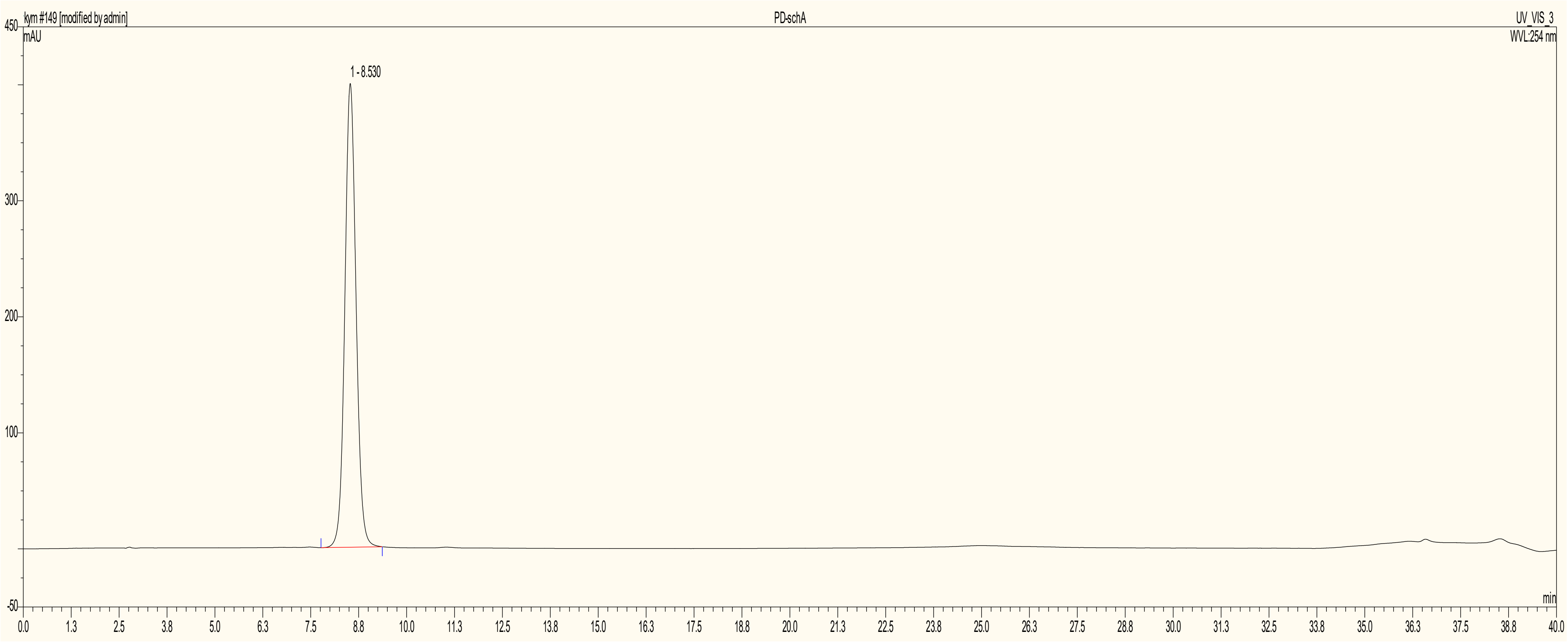

Figure S16. The UHPLC UV chromatogram of Schisandrol A (254 nm)

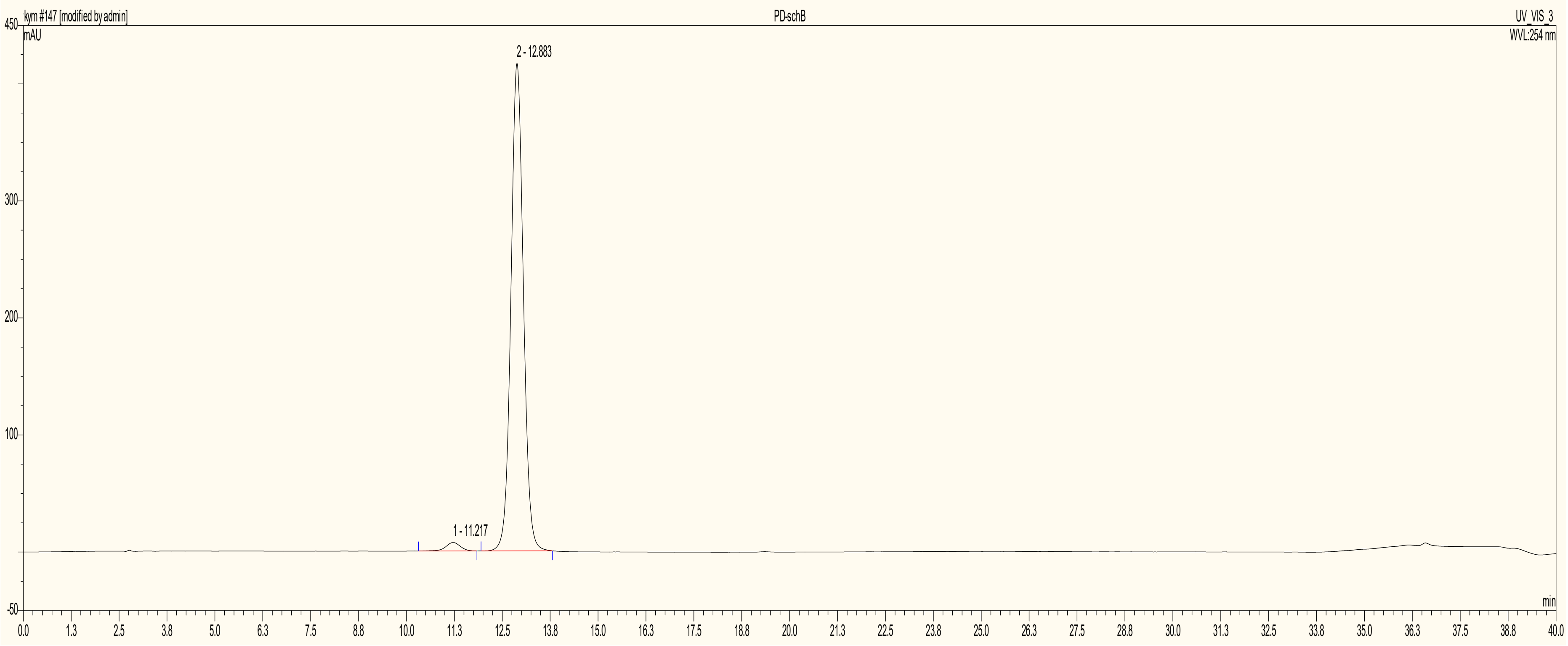

Figure S17. The UHPLC UV chromatogram of Schisandrol B (254 nm)

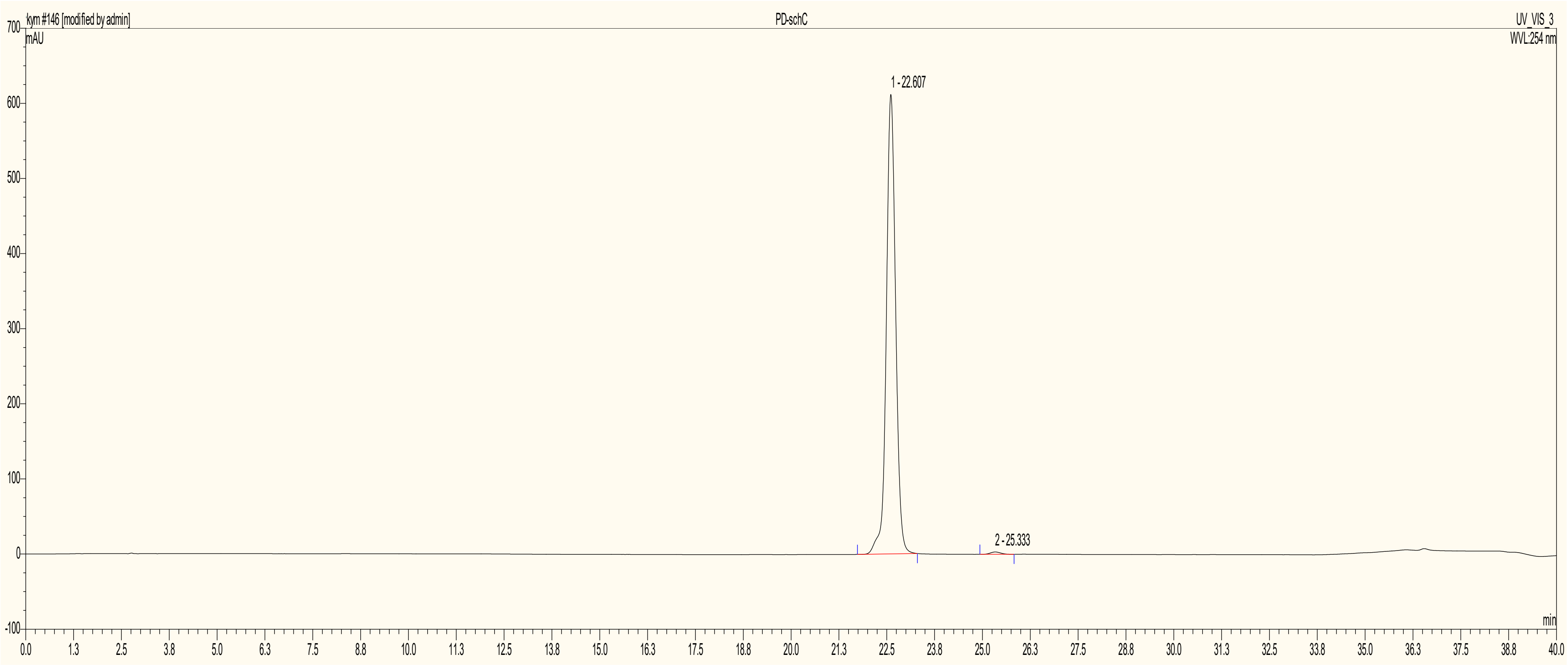

Figure S18. The UHPLC UV chromatogram of Schisandrin C (254 nm)
